# Remodeling of U2-U6 snRNA helix I during pre-mRNA splicing by Prp16 and the NineTeen Complex protein Cwc2

**DOI:** 10.1093/nar/gku431

**Published:** 2014-05-21

**Authors:** Rebecca Hogg, Rogerio Alves de Almeida, Jayalath P.D. Ruckshanthi, Raymond T. O'Keefe

**Affiliations:** Faculty of Life Sciences, The University of Manchester, Oxford Road, Manchester, M13 9PT

## Abstract

Removal of intron regions from pre-messenger RNA (pre-mRNA) requires spliceosome assembly with pre-mRNA, then subsequent spliceosome remodeling to allow activation for the two steps of intron removal. Spliceosome remodeling is carried out through the action of DExD/H-box ATPases that modulate RNA–RNA and protein–RNA interactions. The ATPase Prp16 remodels the spliceosome between the first and second steps of splicing by catalyzing release of first step factors Yju2 and Cwc25 as well as destabilizing U2-U6 snRNA helix I. How Prp16 destabilizes U2-U6 helix I is not clear. We show that the NineTeen Complex (NTC) protein Cwc2 displays genetic interactions with the U6 ACAGAGA, the U6 internal stem loop (ISL) and the U2-U6 helix I, all RNA elements that form the spliceosome active site. We find that one function of Cwc2 is to stabilize U2-U6 snRNA helix I during splicing. Cwc2 also functionally cooperates with the NTC protein Isy1/NTC30. Mutation in Cwc2 can suppress the cold sensitive phenotype of the *prp16-302* mutation indicating a functional link between Cwc2 and Prp16. Specifically the *prp16-302* mutation in Prp16 stabilizes Cwc2 interactions with U6 snRNA and destabilizes Cwc2 interactions with pre-mRNA, indicating antagonistic functions of Cwc2 and Prp16. We propose that Cwc2 is a target for Prp16-mediated spliceosome remodeling during pre-mRNA splicing.

## INTRODUCTION

The removal of intron regions from pre-messenger RNA (pre-mRNA) is catalyzed by the spliceosome, a large RNA–protein complex. The spliceosome is composed of five small nuclear ribonucleoprotein complexes (U1, U2, U4, U5 and U6 snRNPs) and over 100 spliceosome associated proteins (1). The spliceosome also contains five small nuclear RNAs (U1, U2, U4, U5 and U6 snRNAs) associated with the U1, U2, U4, U5 and U6 snRNPs. A number of the snRNAs recognize conserved sequence motifs within the pre-mRNA allowing the spliceosome to identify, then excise, introns in two catalytic steps (2). For example, the U1 snRNA base-pairs with the 5' splice site, the U2 snRNA base-pairs with the branch site and the U5 snRNA interacts with exon sequences (2). Additionally, some snRNAs base-pair with each other to provide essential RNA structures required for the assembly and activation of the spliceosome. Base-pairing of U4-U6 snRNAs occurs during spliceosome assembly, sequestering U6 until it is required to form the spliceosome active site for intron removal. Base-pairing of U2-U6 snRNAs is mutually exclusive with the U4-U6 base-pairing. The U2-U6 base-paired structure, called helix I, was proposed to contribute to the spliceosome active site. Specifically, by comparison with the structure of the functionally related self-splicing group II intron, it was proposed that the U6 AGC triad, which forms part of helix I with U2, establishes tertiary interactions with the U6 ACAGAGA sequence and the U6 internal stem loop (ISL) to produce a spliceosome active site related to that found in group II introns (3). RNA catalysis of splicing has now been proven with recent evidence supporting the role of the U6 snRNA in coordinating divalent metals that stabilize the leaving groups for the two steps of splicing (4). While splicing is RNA catalyzed, the protein components of the spliceosome are essential for positioning and remodeling the RNAs for the two steps of splicing.

The process of spliceosome assembly, intron removal, spliceosome disassembly and spliceosome recycling is highly dynamic, with both the protein composition of the spliceosome and RNA–RNA interactions being remodeled at each step. Remodeling of the spliceosome is facilitated through the action of eight ATPases and one GTPase (5). It is also becoming clear that some of these ATPases contribute to the fidelity of the spliceosome by proofreading specific steps to make sure suboptimal pre-mRNA substrates are not spliced (6–9). One ATPase, Prp2, is the last ATPase to act before spliceosome activation for the first step of splicing (10,11). Prp2 interacts directly with the pre-mRNA, with Prp2 action remodeling protein interactions in the spliceosome (12,13). Specifically, Prp2 action destabilizes the SF3 U2 snRNP complex, the RES complex, Cwc24 and Cwc27 before the first step of splicing while also forming high affinity binding sites for Cwc25 and Yju2, factors required for the first step of splicing (14–20). Recent work has found that Prp2 also destabilizes and proofreads the catalytic RNA core of the spliceosome (21). The ATPase Prp16 contributes to proofreading the first step of splicing before catalyzing rearrangements required for the second step of splicing (22–24). Prp16 was initially identified as a splicing factor that transiently interacts with the spliceosome to promote structural rearrangements that allow the second step of splicing (25,26). Recent work indicates that Prp16 destabilizes Cwc25 and Yju2 from the spliceosome to allow binding of the proteins Prp22, Prp18 and Slu7 required for the second step of splicing (24). Furthermore, Prp16 has been suggested to destabilize the U2-U6 helix I in the spliceosome active site between the first and second step of splicing allowing progression to the second step (23). As Prp16 does not directly interact with U2-U6 helix I, the destabilization of U2-U6 helix I by Prp16 most likely occurs through another protein factor.

During spliceosome assembly a set of proteins associate with the spliceosome to facilitate remodeling and activation. This NineTeen Complex (NTC) of proteins is composed of a protein core associated with Prp19 as well as a number of proteins that have been identified as being associated with these core NTC proteins (27). The interaction of the NTC with the spliceosome is required for the stable binding of the U5 and U6 snRNAs to the pre-mRNA (28). The NTC proteins Yju2 and Cwc25 are required after the Prp2-mediated remodeling of the spliceosome to facilitate the first step of splicing (19). The NTC is linked to the spliceosome active site via the RNA binding protein Cwc2 (29). A zinc finger, RNA recognition motif (RRM) and a unique Torus domain in Cwc2 form a compact folding unit that acts as a multipartite RNA-binding platform (30,31). Cwc2, and its human homolog RBM22, crosslink directly to the U6 snRNA and the pre-mRNA in a similar manner (29,32). Specifically, Cwc2 and RBM22 crosslink to the pre-mRNA just downstream of the 5' splice site (32). Cwc2 and RBM22 also crosslink to the U6 snRNA upstream of the highly conserved ACAGAGA box that interacts with the intron adjacent to the 5' splice site (32). Additionally, Cwc2 and RBM22 crosslink weakly to the U6 snRNA ISL in the B^act^ complex, before the first step of splicing, and then more strongly in the C complex, after the first step of splicing (32). One possible role suggested for these RNA interactions of Cwc2 would be to induce RNA elements of the spliceosome catalytic site into an active conformation (32).

As the functional significance of the Cwc2–RNA interactions are not yet clear, we have combined genetics and crosslinking studies to determine the precise role of Cwc2 during splicing. Using a large scale screen for genetic interactions of Cwc2 with the snRNAs of the spliceosome, we have only found genetic interactions of Cwc2 with the U2 and U6 snRNAs. Most genetic interactions of Cwc2 were localized to the U2-U6 helix I region as well as in the U6 ^47^ACAGAGA^53^ box and U6 ISL. Genetic interactions of Cwc2 with U6 in helix I could be suppressed with dominant compensatory mutations in U2 that restored the U2-U6 helix I base-pairing, indicating that one function of Cwc2 is to stabilize U2-U6 helix I. Prp16 is known to destabilize the U2-U6 helix I between the first and second step of splicing suggesting that Cwc2 may be a target for Prp16 action. In support of this hypothesis, mutation in Cwc2 can suppress the cold sensitive phenotype of the *prp16-302* mutation. In addition, the *prp16-302* mutation destabilizes Cwc2 interaction with the pre-mRNA and stabilizes Cwc2 interaction with the U6 snRNA. Finally, we show that Cwc2 functionally cooperates with Isy1/NTC30, a protein also known to suppress the *prp16-302* mutation. Our data indicate that Cwc2 is a target for Prp16 in the remodeling of helix I during pre-mRNA splicing.

## MATERIALS AND METHODS

### Yeast strains and plasmids

All *Saccharomyces cerevisiae* strains are listed in Supplementary Table 1. The *CWC2*/*snRNA, CWC2*/*PRP16* and *CWC2*/*PRP22* haploid knoc kout strains were constructed from the single *CWC2::kanMX4*/*CWC2* diploid knockout strain Y23907 obtained from EUROSCARF. Individual *snRNA*, *PRP16* or *PRP22* genes were replaced with the *hphNT1* cassette in the *CWC2* diploid knockout strain Y23907. The resulting strains were then transformed with a single pRS416 plasmid containing each of the wild-type genes to complement the two knocked out genes. The diploid double knockout strains were sporulated and haploid tetrads identified that contained the two knocked out genes and the complementing pRS416 plasmid. The haploid CWC2 knockout strain YCWC2KO has been described previously (29). To construct the *CWC2*/*ISY1* and *CWC2*/*ECM2* haploid knockout strains *ISY1* or *ECM2* were replaced with the *hphNT1* cassette in YCWC2KO. To construct a haploid *CWC2*/*CUP1* deletion strain the *CUP1* deletion strain YCL51 was mated with *CWC2/U6* knockout strain CWC2KO/U6KO. The resulting diploid strain was sporulated to identify the haploid strain CWC2KO/CUPKO. The YCWC2TAP strain has been described previously (29). The YCWC2TAP strain was transformed with pRS416-PRP16 or pRS416-*prp16-302*, and then the genomic *PRP16* gene was replaced with the *hphNT1* cassette to produce strains YCWC2TAP-PRP16 and YCWC2TAP-*PRP16-302* for crosslinking. Plasmid pRS413MET15 has been previously described (33).

### Genetic methods

Cwc2/snRNA haploid knockout strains were transformed with pRS413 and pRS415 plasmids alone or these plasmids with different combinations of wild-type or mutant Cwc2 and snRNA genes. Transformants were grown in SD-His-Leu-Ura liquid media, then these cultures were normalized to an OD_600_ of 1. One in five serial dilutions were made and these were spotted onto plates containing 5-fluoroorotic acid (5-FOA) and incubated at 16, 25, 30 and 37°C to search for growth defects of two viable mutations when combined. To suppress the Cwc2 Y34A and F183D growth defect at 30°C with U6 A56C,U57C the plasmids pRS413MET15-U2A27G,U28G or pRS413MET15-U4G62,U63G were transformed into CWC2KO/U6KO together with the Cwc2 mutant and U6 mutant. Transformants were grown in SD-His-Leu-Ura-Met liquid media, then these cultures were normalized to an OD_600_ of 1. One in five serial dilutions were made and these were spotted onto plates containing 5-FOA and incubated at 30°C to determine any suppression. To search for Cwc2 suppressors of the *prp16-302* mutation strain CWC2KO/PRP16KO was transformed with 13 Cwc2 mutant alleles in pRS415 and either wild-type PRP16 or *prp16-302* in pRS413. Transformants were grown in SD-His-Leu-Ura liquid media, then these cultures were normalized to an OD_600_ of 1. One in five serial dilutions were made and these were spotted onto plates containing 5-FOA and incubated at 16, 25, 30 and 37°C to search for Cwc2 suppressors of the *prp16-302* mutation.

### *In vivo* splicing assays

Strain CWC2KO/CUPKO, which contains wild-type *CWC2* on a *URA3* plasmid, was transformed with one of the *ACT1-CUP1* reporters on a *LEU2* plasmid and the Cwc2 mutant Y34A, W37A or F183D on *HIS3* marked plasmids. Transformants were grown on media containing 5-FOA for 3 days to select against the *URA3* marked wild-type *CWC2* plasmid and leave the *HIS3* marked mutant Cwc2 plasmid as the sole source of Cwc2. Yeast strains carrying the specific *ACT1-CUP1* reporter and Cwc2 allele were grown in SD-Leu, then normalized to OD_600_ of 1 for spotting on plates containing different concentrations of CuSO_4_. Plates were photographed after 3 days at 30°C.

### RNA production, *in vitro* splicing, crosslinking, primer extension and western blotting

Plasmid pBS-Actin was used to produce *ACT1* pre-mRNA and biotin containing *ACT1* pre-mRNA by *in vitro* transcription (29). The UTP:Biotin-16-UTP ratio in the transcription reaction for biotin-containing pre-mRNA was 50:1. Crosslinking to detect Cwc2-U6 snRNA interactions and Cwc2-pre-mRNA interactions was carried out as follows. Splicing reactions were performed in duplicate (one for crosslinking and one for negative control no crosslinking) in 250 μl total volume containing 100 μl of yeast whole cell extract, 50 μl 5× splice buffer (10 mM ATP, 12.5 mM MgCl_2_ and 300 mM KPO_4_ pH 7), 25 μl 30% PEG and 2 μl of 1 μM *ACT1* pre-mRNA or biotin containing *ACT1* pre-mRNA. Wild-type and dominant negative proteins were added to splicing reactions at a concentration of 8.5 ng/μl and incubated at 23°C for 20 min before pre-mRNA was added to initiate splicing for 35 min. For splicing of extracts from strains YCWC2TAP-PRP16 and YCWC2TAP-*PRP16-302*, reactions were incubated at 18°C for 55 min. Aliquots of 25 μl were placed on a parafilm covered aluminium block on ice and crosslinked three times using a Stratagene Stratalinker set at 4111 μJ/cm^2^ × 100.

To select U6 snRNA from crosslinked splicing reactions, 200 μl Dynabeads M-270 Streptavidin was washed three times with 400 μl 1× binding buffer (10 mM Tris HCl pH 7.5, 0.5 M NaCl, 0.5% SDS and 0.1 mM EDTA), and then re-suspended in 200 μl of 1× binding buffer. To the washed magnetic beads, 20 μl of 10 μM biotinylated oligo nucleotide complementary to the U6 snRNA (U6 BioTEG 5'AAAACGAAATAAATCTCTTTG) was added and incubated at room temperature for 15 min. Magnetic beads were then washed three times with 800 μl 1× binding buffer and re-suspended in 200 μl of 1× binding buffer. To select biotin containing *ACT1* pre-mRNA from crosslinked splicing reactions, 200 μl Dynabeads M-270 Streptavidin were washed three times with 400 μl 1× binding buffer and re-suspended in 200 μl 1× binding buffer.

Crosslinked splicing reactions as well as negative control reactions (not crosslinked samples) were each mixed with 200 μl of the prepared magnetic beads and 250 μl of 2× binding buffer. Samples were incubated for 1 h at room temperature and mixed twice during the incubation period. Magnetic beads were washed three times with 900 μl 1× binding buffer, then eluted with 800 μl elution buffer (10 mM Tris HCl pH 7.5 and 0.1 mM EDTA). The binding buffer contains 0.5 M NaCl and 0.5% SDS (see above), which provides stringent conditions to only allow selection of proteins that directly crosslink to the RNA. The sample was then divided into two parts, 200 μl for primer extension analysis of selected RNAs and 600 μl for western blotting to detect crosslinked Cwc2.

To isolate RNA from the 200 μl part of the sample, 200 μl citrate buffered phenol:chloroform:isoamyl alcohol (25:24:1) was added. The mixture was vortexed for 2 min, then centrifuged at 13 000 rpm for 2 min to separate the two phases. Next, 190 μl of the upper phase was transferred to a new tube and mixed with 19 μl 3 M NaOAc pH 5.3, 2 μl *Escherichia coli* tRNA (10 mg/ml) and 475 μl ice-cold 100% ethanol. Samples were then incubated at −20°C to precipitate the RNA.

For primer extension, precipitated RNA was re-suspended in 10 μl of 50 mM Tris HCl pH 8.5, 8 mM MgCl_2_, 30 mM KCl, 1 mM dithiothreitol and 50 picomoles of ^32^P labelled primer. For detection of the U6 snRNA primer U6RTAll (5'-TCATCCTTATGCAGGG) was used. For detection of *ACT1* pre-mRNA primer ISeq (5'-GAAATCTCTCGAGCAATTGGG) was used. This mixture was heated to 90°C and cooled slowly to 41°C. To this was added 10 μl of primer extension mix (50 mM Tris HCl pH 8.5, 8 mM MgCl_2_, 30 mM KCl, 1 mM dithiothreitol, 0.7 mM each dNTP), 20 units RNasin (Promega) and 3 units AMV reverse transcriptase (Roche). The mixture was incubated at 41°C for 30 min, then the volume was brought to 200 μl with 180 μl of splicing diluent (300 mM NaOAc pH 5.3, 1 mM EDTA, 0.1% SDS, 25 μg/ml *E. coli* tRNA). An equal volume of Tris pH 8 buffered phenol:chloroform:isoamyl alcohol (25:24:1) was added and the mixture vortexed then centrifuged to separate the phases. The upper phase was removed and 2.5 volumes of 100% ethanol was added to precipitate the primer extension products. The precipitated primer extension products were re-suspended in formamide loading buffer (95% formamide, 10 mM EDTA, 0.025% w/v bromophenol blue, 0.025% w/v xylene cyanol FF) then loaded on a 6% polyacrylamide/7 M urea gel to separate the primer extension products. Dried gels were exposed to a phosphorimaging screen for quantification of primer extension products with a BioRad FX phosphorimager using ImageQuant software.

For western blotting, the 600 μl part was mixed with 6 μl of RNase cocktail (Ambion) and incubated at 37°C for 45 min. The magnetic beads were captured, then the sample removed from the beads and transferred to a new tube with 150 μl freshly made 100% trichloroacetic acid and incubated for 20 min on ice. Samples were centrifuged at 13 000 rpm at 4°C for 15 min and the resulting protein pellet was washed with 800 μl ice-cold acetone. The pellet and acetone were centrifuged for 5 min at 13 000 rpm at 4°C, the acetone was removed and the pellet re-suspended in 15 μl SDS-sample buffer (50 mM Tris-HCl pH 6.8, 2% SDS, 0.005% bromophenol blue, 10% glycerol, 50 mM DTT) with 2 μl 1 M Tris HCl pH 8. The sample was loaded on a 10% SDS-PAGE and proteins separated by electrophoresis at 200 V for 1 h. Proteins were transferred to a nitrocellulose membrane with a semi-dry transfer apparatus (BioRad) at 10 V for 1 h. The membrane was blocked for 1 h with 5% non-fat milk in TBS-T (10 mM Tris-HCl pH 7.4, 150 mM NaCl, 0.05% Tween 20). Primary polyclonal rabbit anti-TAP antibody (1:2000 dilution, AFCAB1001; Thermo Scientific) was diluted with 5% non-fat milk in TBS-T and incubated with the membrane overnight with shaking at 4°C. The membrane was washed six times with distilled water and washed once with TBS-T for 5 min. The membrane was incubated with goat anti-rabbit secondary antibody (1:5000 dilution, 926-32211; IRDye 800CW - Li-Cor Biosciences) for 1 h at room temperature, then washed six times with distilled water and washed once with TBS-T for 5 min. Proteins were visualized using a LI-COR Odyssey instrument and signals were quantified using the Image Studio software version 2.1.

Expression and purification of His-tagged wild-type Prp16 and Prp16-D473A from bacteria was according to standard procedures. The wild-type Prp16 bacteria expression plasmid pET22b-Prp16 was a gift from Jana Schmitzova, and the Prp16-D473A mutation was made in this plasmid. Expression and purification of wild-type Prp2 and Prp2-LAT dominant negative mutant protein was as previously described (34).

## RESULTS

### Cwc2 interacts genetically with the U6 and U2 snRNAs

**Figure 1.**
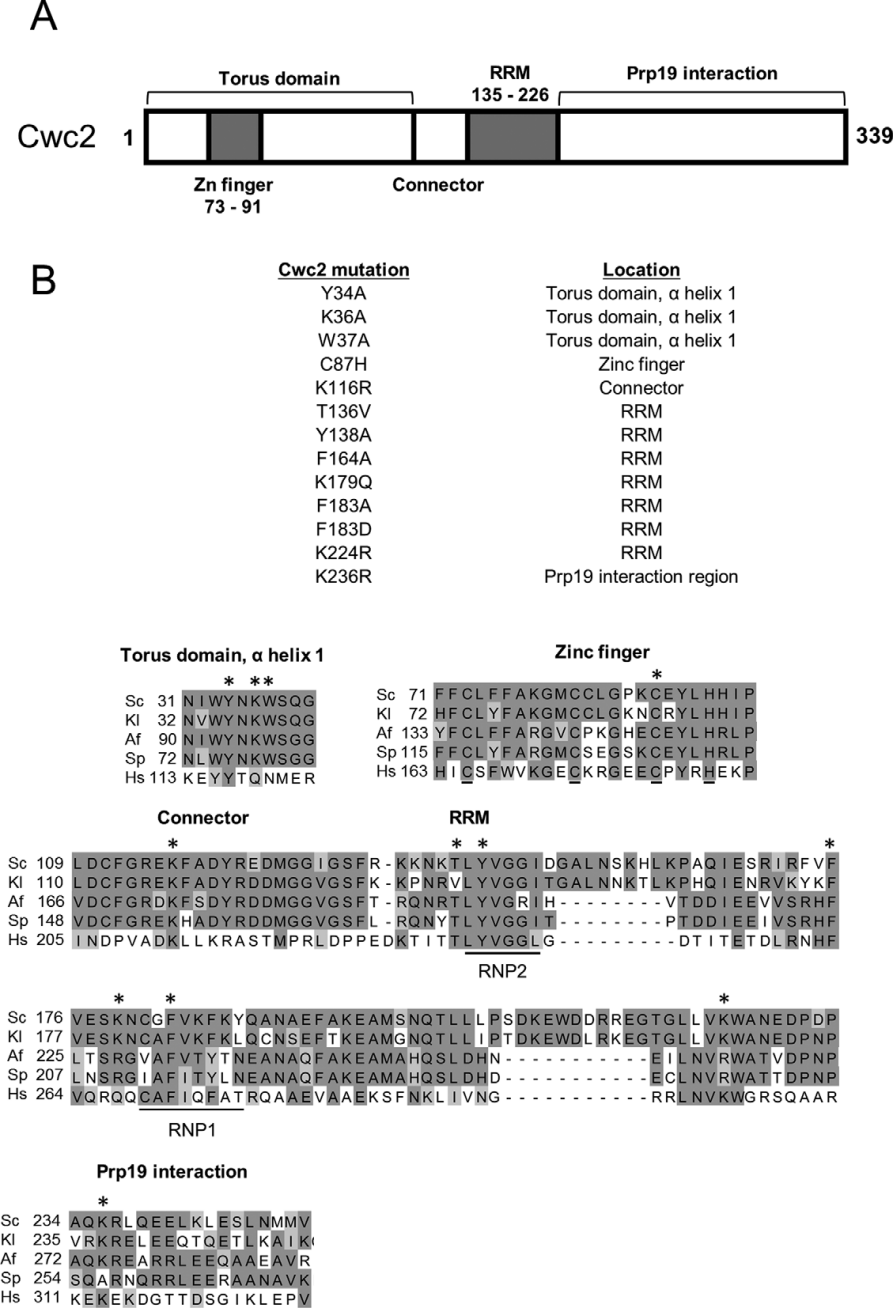
Domain structure and viable mutations in Cwc2. (**A**) The location of known protein motifs in the *Saccharomyces cerevisiae* Cwc2 protein are depicted including the zinc (Zn) finger, Torus domain, connector, RNA recognition motif (RRM) and Prp19 interaction domain. (**B**) Viable mutations in Cwc2 used for genetic interaction analysis. The locations of each mutation in the Cwc2 protein structure are described to the right of each mutation. The amino acid sequence of Cwc2 from *Saccharomyces cerevisiae* (Sc, NCBI-GI: 6319992) was aligned with orthologs from *Kluyveromyces lactis* (Kl, NCBI-GI: 50305857), *Aspergillus fumigatus* (Af, NCBI-GI: 70998470), *Schizosaccharomyces pombe* (Sp, NCBI-GI: 19114249) and *Homo sapiens* (Hs, NCBI-GI: 8922328). The locations of the Cwc2 mutants are identified with asterisks above the selected regions of the alignment.

Cwc2 has a protein domain structure consisting of a Torus domain, zinc (Zn) finger, RRM and a C-terminal domain that interacts with Prp19 (Figure 1A). Crosslinking has revealed that Cwc2 interacts directly with the U6 snRNA and pre-mRNA (29,32), however, the functional significance of these interactions is not clear. To investigate how Cwc2 functionally interacts with the snRNAs of the spliceosome we carried out an extensive genetic screen with 54 viable snRNA mutations in the U2, U4, U5 and U6 snRNAs (Supplementary Figures S1–S4) and 13 viable mutants in Cwc2 (Figure 1B) to search for functional interactions between Cwc2 and these snRNAs. Six of the Cwc2 mutants (Y34A, W37A, C87H, T136V, Y138A and F183D) were viable mutants in highly conserved regions of Cwc2 (Figure 1B) that we had identified in a previous study (29). The remaining viable Cwc2 mutants (K36A, K116R, F164A, K179Q, F183A, K224R and K236R) were designed for this study to provide a wider range of mutations in highly conserved amino acids (Figure 1B).

Four haploid yeast strains were constructed where Cwc2 and either the U2, U4, U5 or U6 snRNA were deleted and the deletions complemented with a *URA3* plasmid containing the wild-type *CWC2* gene and wild-type snRNA gene. These strains were then transformed with each of the thirteen Cwc2 mutants in combination with each of the 54 snRNA mutations as well as the appropriate controls. Growth of each strain on 5-FOA selects against the *URA3* plasmid containing the wild-type genes allowing the growth of the two viable mutations to be assessed together.

**Figure 2.**
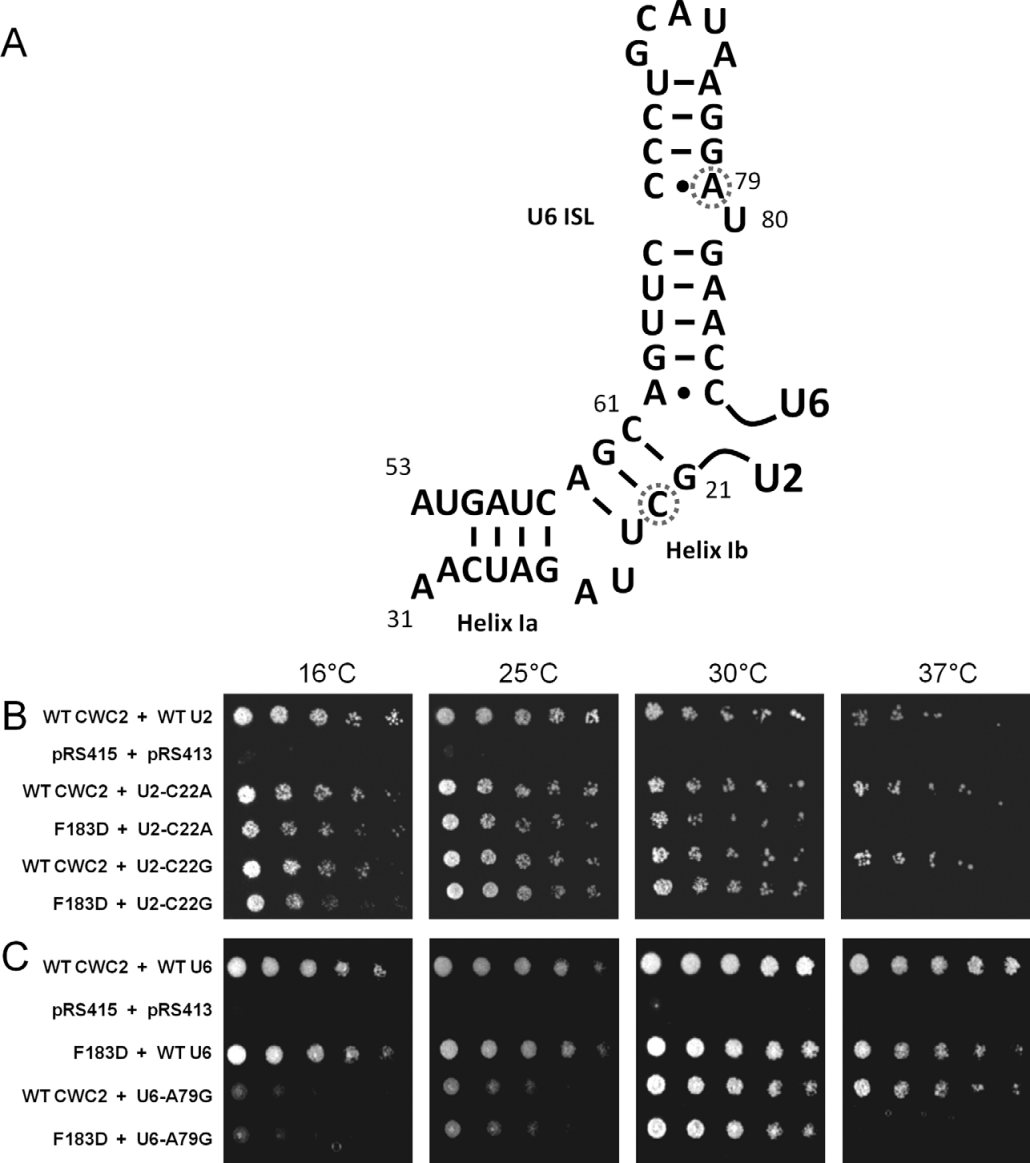
Cwc2 F183D is lethal with U2-C22 substitutions and U6-A79G at 37°C. (**A**) The secondary structure of U2-U6 helix I and the U6 ISL are displayed with the location of U2-U22 and U6-A79 noted by a dashed circle. (**B**) The plasmid shuffle assay was performed in the CWC2KO/U2KO strain containing the wild-type *CWC2* and *U2* genes on a *URA3* marked plasmid. The strain was co-transformed with mutant Cwc2 and U2 alleles on *LEU2* and *HIS3* marked plasmids, respectively. One in five serial dilutions of yeast, with an initial OD_600_ of 1, were grown on plates containing 5-FOA at 16, 25, 30 and 37°C for 3–5 days (5 days for plates at 16 and 25°C), selecting against the wild-type *URA3* plasmid to determine the growth phenotype of combining two viable mutations. (**C**) The plasmid shuffle assay was performed in the CWC2KO/U6KO strain containing the wild-type *CWC2* and *U6* genes on a *URA3* marked plasmid. The strain was co-transformed with mutant Cwc2 and U6 alleles on *LEU2* and *HIS3* marked plasmids, respectively. One in five serial dilutions of yeast, with an initial OD_600_ of 1, were grown on plates containing 5-FOA at 16, 25, 30 and 37°C for 3–5 days (5 days for plates at 16 and 25°C), selecting against the wild-type *URA3* plasmid to determine the growth phenotype of combining two viable mutations.

**Figure 3.**
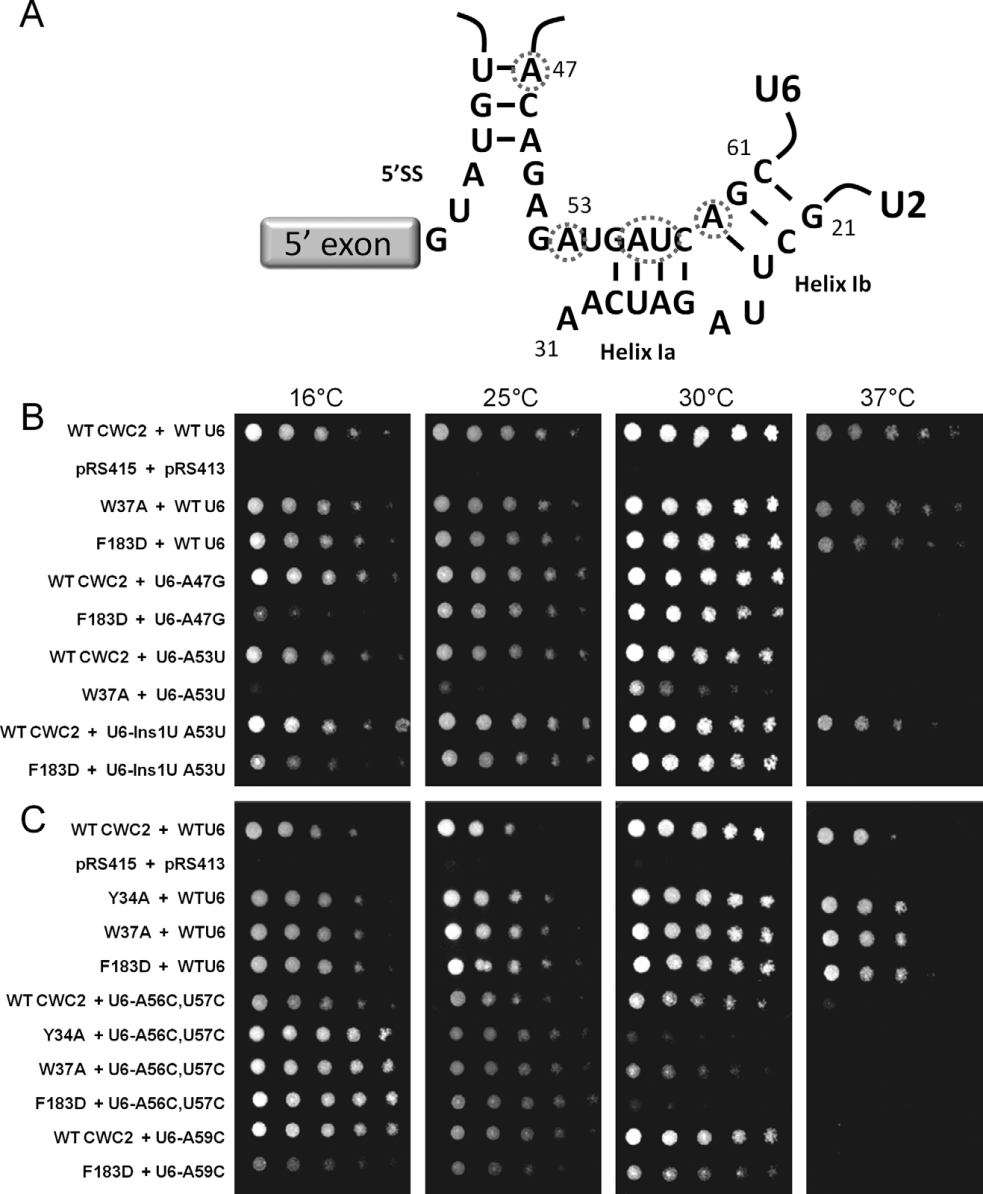
Viable Cwc2 mutations are lethal and sick with mutations in the U6 AGAGAGA and U2-U6 helix I (**A**) Interaction of U6 with the 5' splice site and the structure of U2-U6 helix I are displayed with the location of U6-A47, U6-A53, U6-A56, U6-A57 and U6-A59 noted by dashed circles. (**B** and **C**) The plasmid shuffle assay was performed in the CWC2KO/U6KO strain containing the wild-type *CWC2* and *U6* genes on a *URA3* marked plasmid. The strain was co-transformed with mutant Cwc2 and U6 alleles on *LEU2* and *HIS3* marked plasmids respectively. One in five serial dilutions of yeast, with an initial OD_600_ of 1 were grown on plates containing 5-FOA at 16, 25, 30 and 37°C for 3–5 days (5 days for plates at 16 and 25°C), selecting against the wild-type *URA3* plasmid to determine the growth phenotype of combining two viable mutations.

Of the 702 interactions investigated, only nine combinations of Cwc2 and snRNA viable mutations displayed a growth defect at 16°C, 25°C, 30°C or 37°C. No genetic interactions were found between Cwc2 and any of the U4 or U5 snRNA mutants (data not shown). The Cwc2 RRM mutation F183D was lethal at 37°C combined with the U2-C22A and U2-C22G mutation in U2-U6 helix Ib (Figure 2A and B). The remaining genetic interactions were only found between Cwc2 mutations Y34A, W37A, F183D and the U6 snRNA. Three specific regions of the U6 snRNA displayed genetic interactions with the Y34A, W37A and F183D mutations in Cwc2. The first region of U6 to display genetic interactions with Cwc2 was the U6 ISL. The mutation U6-A79G in the U6 ISL was lethal at 37°C with the Cwc2 F183D mutation (Figure 2C). The second region of U6 to display genetic interactions with Cwc2 was the U6 ^47^ACAGAGA^53^ box. The U6-A47G mutation in the ^47^ACAGAGA^53^ box was lethal at 16°C combined with the Cwc2 F183D mutation (Figure 3B). The U6-A53U mutation, also in the ^47^ACAGAGA^53^ box, was lethal at 16°C and 25°C and sick at 30°C when combined with Cwc2 W37A (Figure 3B). Insertion of 1 uridine after U6-A53 (U6-Ins1 A53) was sick at 16°C and lethal at 37°C when combined with Cwc2 F183D (Figure 3A and B). The third region of U6 to display genetic interactions with Cwc2 was helix I. The helix Ia mutation A56C,U57C was lethal at 30°C with Cwc2 mutations Y34A and F183D (Figure 3A and C). The helix Ib mutation U6-A59C was sick at 16°C with Cwc2 F183D (Figure 3A and C). Overall these genetic data indicate that Cwc2 functionally interacts with the three RNA regions implicated in forming the active site of the spliceosome.

### Cwc2 stabilizes U2-U6 helix I

**Figure 4.**
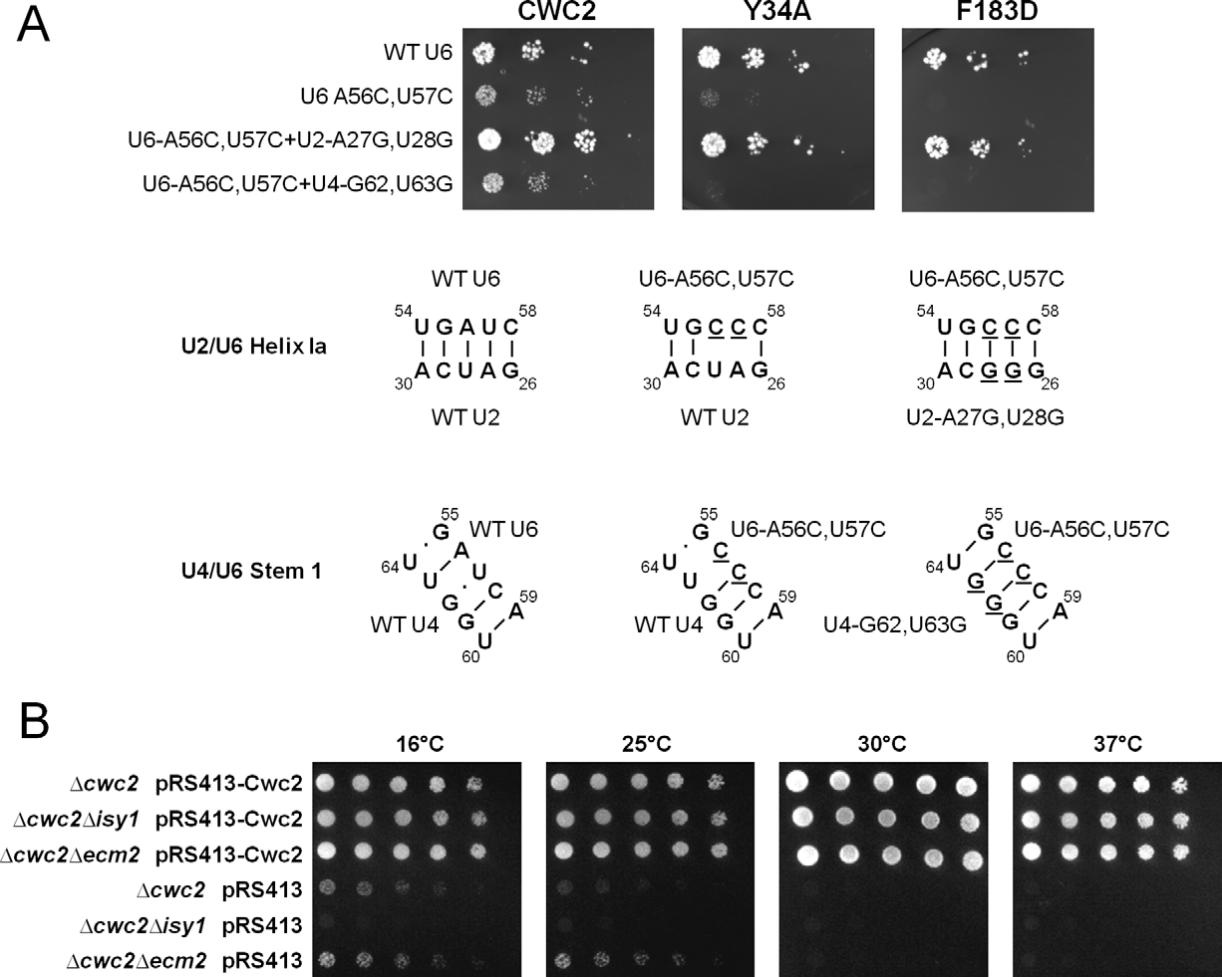
Synthetic lethal interactions between Cwc2 Y34A or Cwc2 F183D alleles with U6-A56C,U57C can be rescued by mutations in U2 that restore U2/U6 helix Ia. (**A**) Yeast strains with synthetic lethal interactions between Cwc2 Y34A or Cwc2 F183D with the U6-A56C,U57C allele can be rescued with the transformation of a dominant U2-A27G,U28G plasmid that restores base-pairing in U2/U6 helix Ia. Restoring base-pairing of U4/U6 stem I with the U4-G62,U63G plasmid does not rescue synthetic lethality. The CWC2KO/U6KO strain containing wild-type *CWC2* and *U6* genes on a *URA3* marked plasmid was used for the plasmid shuffle assay. The strain was co-transformed with mutant Cwc2, U6 and U2 or U4 alleles on *LEU2*, *HIS3* and *MET15* marked plasmids, respectively. One in five serial dilutions of yeast, with an initial OD_600_ of 1, were grown on plates containing 5-FOA at 30°C for 3 days to select against the wild-type *URA3* plasmid. The bottom of panel in (A) shows the RNA structures of regions within the U2/U6 helix Ia and the U4/U6 stem 1. Underlined letters denote changed nucleotides. (**B**) ΔCwc2 is synthetic lethal with deletion of the non-essential NTC protein Isy1. The plasmid shuffle assay was performed in the CWC2KO, CWC2/ISY1 KO and CWC2/ECM2 KO haploid yeast strains, containing wild-type *CWC2* on a *URA3* marked plasmid. Strains were transformed with either wild-type *CWC2* on a *HIS3* marked plasmid as a control or with empty *HIS3* plasmid alone. One in five serial dilutions of yeast with an initial OD_600_ of 1 were grown on media containing 5-FOA at 16, 25, 30 and 37°C for 3–5 days (5 days for plates at 16 and 25°C). The CWC2KO strain is viable at 16 and 25°C, however the double knockout strain *Δcwc2Δisy1* is lethal. This synthetic lethality is not seen with another NTC protein Ecm2, as the *Δcwc2Δecm2* double mutant shows growth at 16 and 25°C.

Genetic interactions of Cwc2 with mutations in U6 that destabilize the U2-U6 helix I suggest that Cwc2 may be involved in stabilizing U2-U6 helix I. However, the U6 residues that are mutated also form interactions with the U4 snRNA in the tri-snRNP. If the genetic interactions between Cwc2 and U6 indeed reflect a role of Cwc2 in stabilizing U2-U6 helix I then restoring U2-U6 base-pairing should suppress the growth defect. To this end we introduced into strains the Cwc2 mutant Y34A or F183D, U6 mutant A56C,U57C and an additional copy of U2 containing the A27G,U28G mutation that would restore base-pairing in helix I. As predicted, restoring U2-U6 base-pairing suppressed the growth defect of Cwc2 mutant Y34A or F183D with U6-A56C,U57C (Figure 4A). On the other hand, introducing an additional copy of U4 containing the U63G mutation that restored U4-U6 base-pairing did not suppress the growth defect of Cwc2 mutant Y34A or F183D with U6-A56C,U57C (Figure 4A). Therefore, suppression of the genetic interaction that Cwc2 mutant Y34A or F183D has with U6-A56C,U57C indicates that one function of Cwc2 is to stabilize U2-U6 helix I.

A prediction of a factor that stabilizes an RNA–RNA interaction is that at lower temperatures RNA–RNA interactions would be more stable and may abrogate the need for a stabilizing factor. This is in fact the case for Cwc2. We have observed that deletion of Cwc2 is lethal at 37°C and 30°C. However, when a strain with a Cwc2 deletion is grown at the lower temperatures of 25°C and 16°C, the strain begins to show increased viability as the temperature is dropped, but this viability does not approach wild-type levels (Figure 4B). This suppression of growth defect at lower temperatures in the Cwc2 deletion strain again supports a role for Cwc2 in stabilizing RNA–RNA interactions.

Cwc2 is not the only protein of the NTC to show genetic interactions with U6. Deletion of the non-essential NTC protein Isy1/NTC30 displays a growth defect when combined with the U6-U57A mutation in helix I. It was suggested that Isy1, as part of the NTC, may engage with U2-U6 helix I and modulate the transition of the spliceosome between the first and second step conformational states (35). Therefore, Isy1 may also be involved in stabilizing U2-U6 helix I with Cwc2. We tested this hypothesis by combining deletion of Cwc2 and Isy1 at 25°C and 16°C, temperatures that support some growth of the Cwc2 deletion. We found that combining Cwc2 and Isy1 deletions prevented cell growth (Figure 4B). Deletion of Cwc2 and Ecm2, another non-essential NTC protein that contains a zinc finger and RRM like Cwc2 (36), still allowed growth of the Cwc2 deletion strain at low temperatures and this growth appeared to be slightly improved (Figure 4B). The synergistic effect of Cwc2 and Isy1 deletion on cell growth indicates that these NTC proteins may cooperate in stabilizing U2-U6 helix I.

### Cwc2 mutants display dichotomy in their influence on *in vivo* splicing fidelity

**Figure 5.**
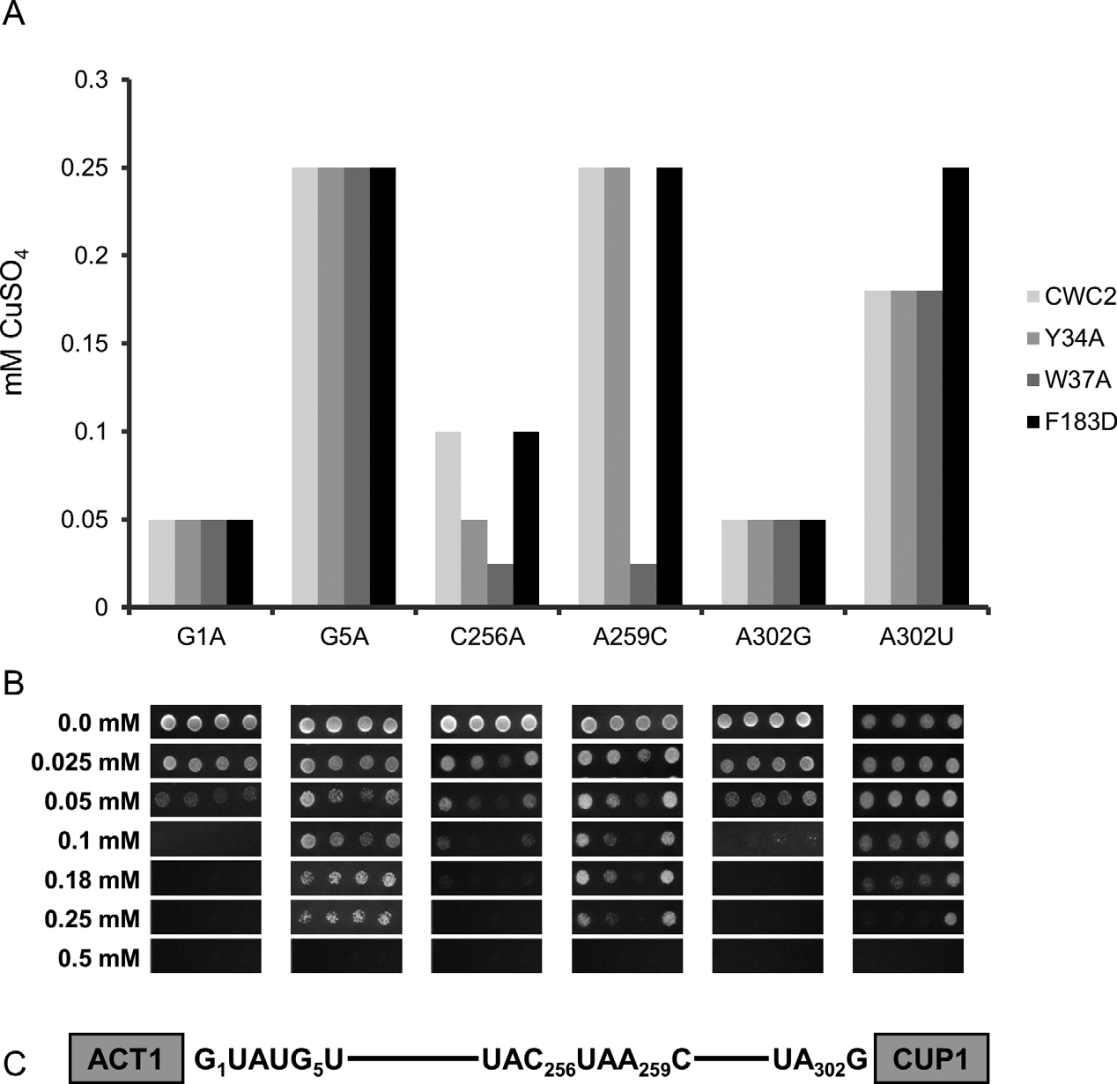
Cwc2 alleles display dichotomy in their influence on *in vivo* splicing. (**A**) The yeast strain CWC2KO/CUPKO, which contains wild-type Cwc2 on a *URA3* marked plasmid, was transformed with one of the *ACT1-CUP1* reporters indicated below each graph and one Cwc2 mutant on the *HIS3* marked plasmid indicated in the box on the right. Strains were grown on media containing 5-FOA for 3 days to select against the *URA3* marked wild-type plasmid and leave the *HIS3* marked mutant Cwc2 plasmid as the sole source of Cwc2. One in five serial dilutions of strains grown in SD-Leu were spotted on plates containing different concentrations of CuSO_4_ for 5 days at 30°C. The bar graph represents the highest concentration of CuSO_4_ tolerated by the yeast after 5 days. (**B**) Copper growth phenotype of the Cwc2 Y34A, W37A and F183D mutants. Yeast cultures carrying the specific *ACT1-CUP1* reporter and Cwc2 allele were normalized to OD_600_ of 1 and spotted on plates containing CuSO_4_ at the indicated concentrations. Plates were photographed after 3 days at 30°C (**C**) Schematic representation of the *ACT1-CUP1* reporter with the position number of the intron mutations used indicated.

To further support the apparent synergy in function of Cwc2 and Isy1, we decided to investigate the *in vivo* splicing fidelity of Cwc2 mutants with the widely used *ACT1-CUP1* reporter (37). Deletion of Isy1 is known to exacerbate splicing of the *ACT1-CUP1* reporter carrying a branch nucleotide A259C mutation and to decrease accuracy of 3' splice site selection by increasing splicing of the A302G and A302U 3' splice site mutant reporters (35). We tested the *in vivo* splicing fidelity of the Cwc2 mutants Y34A, W37A and F183D that displayed genetic interactions with the U2 and U6 snRNAs using a range of *ACT1-CUP1* reporters. Mutation in Cwc2 displayed the same *in vivo* splicing fidelity phenotype as that observed with Isy1 deletion, exacerbation of branch site mutant reporters C256A and A259C as well as increased splicing of the 3' splice site mutant reporter A302U (Figure 5). Interestingly, the Cwc2 Y34A and W37A mutations only exacerbated splicing of branch site mutant reporters with Y34A influencing the C256A reporter and W37A influencing both the C256A and A259C reporters (Figure 5). On the other hand, the Cwc2 F183D mutation only increased splicing of the 3' splice site mutant reporter A302U. This dichotomy of splicing phenotype for different Cwc2 mutants provides evidence that the Torus domain and RRM have different functions.

### Mutation in Cwc2 suppresses the *prp16-302* mutation

**Figure 6.**
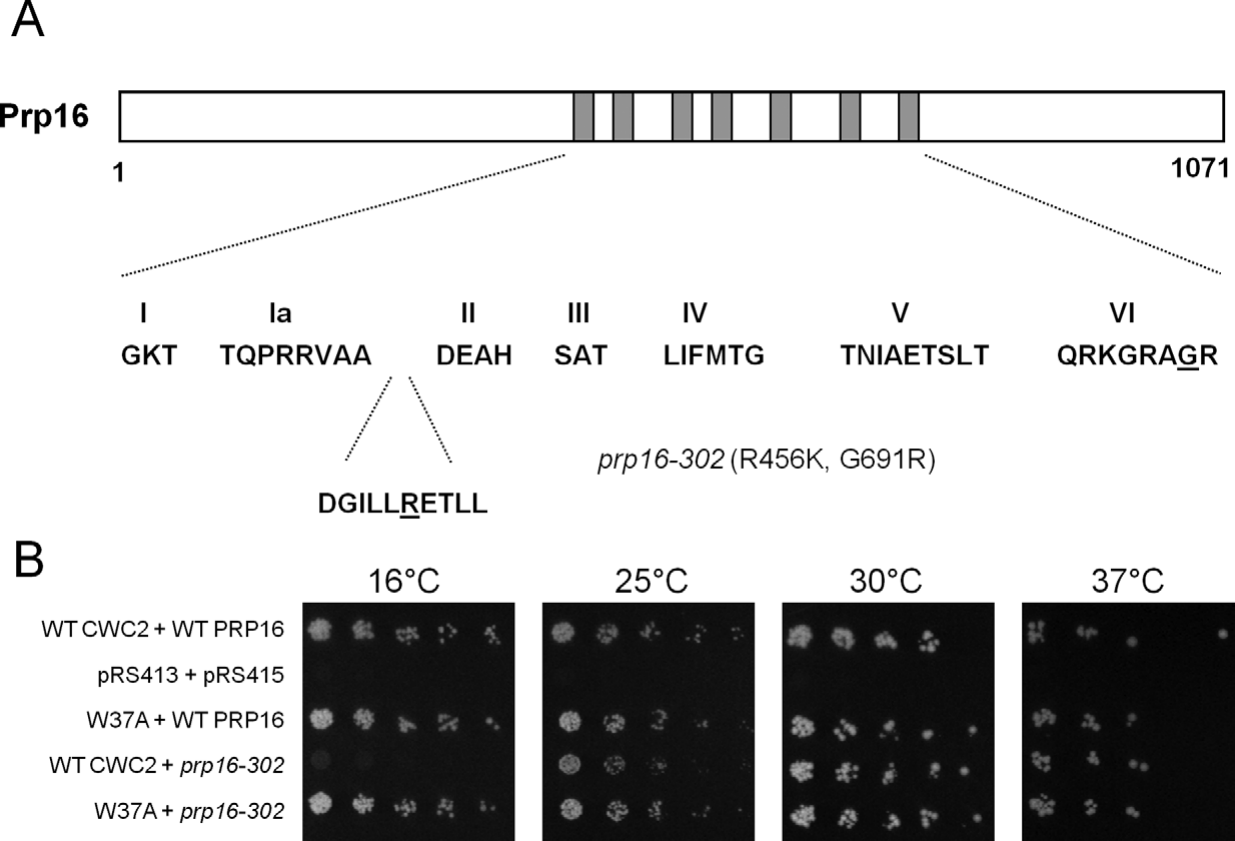
Cwc2 W37A suppresses the *prp16-302* cold sensitive growth defect at 16°C. (**A**) Schematic diagram of the Prp16 protein. The seven conserved domains that define DExD/H box proteins are shown in grey. The sequences of these motifs within Prp16 are shown. The *prp16-302* mutant carries two mutations. The R456K mutation is located in a highly conserved residue located in between domain Ia and II. The G691R mutation is located in the conserved VI domain. (**B**) Genetic suppression of *prp16-302* by Cwc2 W37A. The plasmid shuffle assay was performed in the CWC2KO/PRP16KO strain containing the wild-type CWC2 and PRP16 genes on a *URA3* marked plasmid. The strain was co-transformed with mutant Cwc2 and Prp16 alleles on *LEU2* and *HIS3* marked plasmids, respectively. One in five serial dilutions of yeast, with an initial OD_600_ of 1, were grown on plates containing 5-FOA at 16, 25, 30 and 37°C for 3 days, except for strains grown at 16°C which were left for 5 days.

Prp16 proofreads the first step of splicing, then remodels the spliceosome before the second step of splicing by releasing Cwc25 and Yju2 as well as destabilizing helix I (22–24). As Cwc2 stabilizes helix I and displays synergism with Isy1, a protein known to interact genetically with Prp16 (35), we looked to see if any of the Cwc2 mutations suppressed the *prp16-302* mutation. The *prp16-302* mutation is lethal at 16°C and is the most cold sensitive of the Prp16 mutants due to the double substitutions R456K and G691R located within the ATPase domain (35,38,39) (Figure 6A). At the restrictive temperature of 16°C, the *prp16-302* mutation causes a block to the second step of splicing and stalls the release of Prp16 from the spliceosome *in vitro* (35). The cold sensitive phenotype is the result of inefficient ATPase activity required to remodel the spliceosome from the first to the second step conformation. The increase in thermal energy at higher temperatures bypasses this weakened ATPase activity. We tested the 13 viable mutations in Cwc2 for their ability to suppress the growth defect of *prp16-302* at 16°C. Only the W37A mutation in alpha helix 1 of the Torus domain in Cwc2 suppressed the *prp16-302* at 16°C (Figure 6B). This suppression of the *prp16-302* mutation by mutation in Cwc2 indicates that impairing Cwc2-dependent stabilization allows the spliceosome to bypass the requirement for a fully functional Prp16 and identifies a functional link between Cwc2 and Prp16. To test the specificity of suppression of Cwc2 mutation to Prp16 we analyzed whether the 13 viable mutations in Cwc2 could suppress five cold sensitive mutations in Prp22 (Supplementary Figure S5), another ATPase of the spliceosome required after the Prp16 step. No Cwc2 mutations suppressed the cold sensitive mutations in Prp22 (data not shown) supporting the specificity of Cwc2 suppression of the *prp16-302* mutation.

### Mutation in Prp16 influences Cwc2 interaction with the U6 snRNA and pre-mRNA

**Figure 7.**
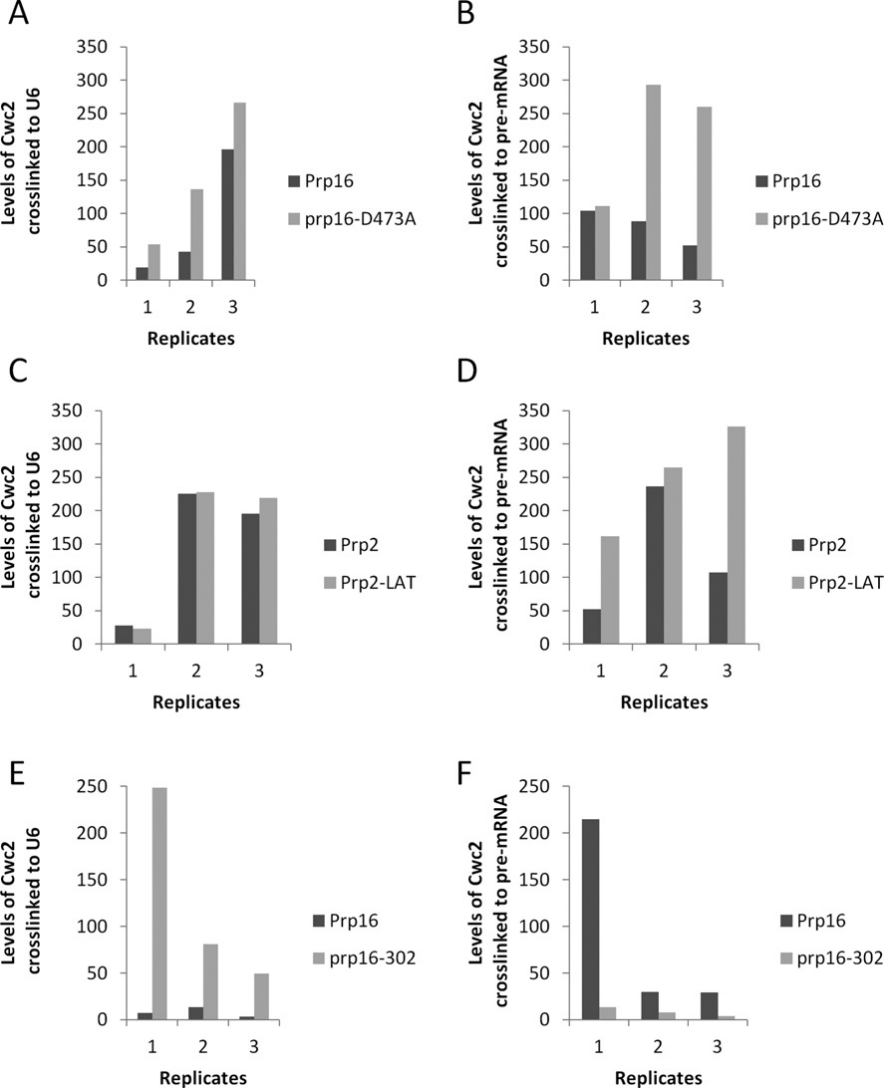
Crosslinking of Cwc2 to the U6 snRNA or pre-mRNA in the presence of Prp16 D473A, Prp2-LAT or *prp16-302*. (**A**) Cwc2-U6 crosslinks in the presence of wild-type Prp16 or Prp16 D473A. Yeast whole cell extract was made from yeast strains containing TAP-tagged Cwc2. Splicing reactions with *ACT1* pre-mRNA were UV irradiated before selection with a biotinylated oligonucleotide complementary to U6 snRNA, under denaturing conditions. Selected proteins were separated by SDS-PAGE and Cwc2-TAP was detected with anti-TAP antibody by immunoblotting. Levels of Cwc2 crosslinked to U6 were quantified in three separate experiments and normalized to the amount of U6 selected from the reactions (Supplementary Figure S7). Unpaired *t*-test with Welch's correction of the three replicates (*P* = 0.8927) (**B**) Cwc2-pre-mRNA crosslinks in the presence of wild-type Prp16 or Prp16 D473A. Yeast whole cell extract was made from yeast strains containing TAP-tagged Cwc2. Splicing reactions with biotin-containing *ACT1* pre-mRNA were UV irradiated before selection with streptavidin paramagnetic beads, under denaturing conditions. Selected proteins were separated by SDS-PAGE and Cwc2-TAP was detected with anti-TAP antibody by immunoblotting. Levels of Cwc2 crosslinked to pre-mRNA were quantified in three separate experiments and normalized to the amount of U6 selected from the reactions (Supplementary Figure S8). Unpaired *t*-test with Welch's correction of the three replicates (*P* = 0.1442) (**C**) Cwc2-U6 crosslinks in the presence of wild-type Prp2 or Prp2 LAT. Yeast whole cell extract was made from yeast strains containing TAP-tagged Cwc2. Splicing reactions with *ACT1* pre-mRNA were UV irradiated before selection with a biotinylated oligonucleotide complementary to U6 snRNA, under denaturing conditions. Selected proteins were separated by SDS-PAGE and Cwc2-TAP was detected with anti-TAP antibody by immunoblotting. Levels of Cwc2 crosslinked to U6 were quantified in three separate experiments and normalized to the amount of U6 selected from the reactions (Supplementary Figure S9). Unpaired *t*-test with Welch's correction of the three replicates (*P* = 0.9177). (**D**) Cwc2-pre-mRNA crosslinks in the presence of wild-type Prp2 or Prp2 LAT. Yeast whole cell extract was made from yeast strains containing TAP-tagged Cwc2. Splicing reactions with biotin-containing *ACT1* pre-mRNA were UV irradiated before selection with streptavidin paramagnetic beads, under denaturing conditions. Selected proteins were separated by SDS-PAGE and Cwc2-TAP was detected with anti-TAP antibody by immunoblotting. Levels of Cwc2 crosslinked to pre-mRNA were quantified in three separate experiments and normalized to the amount of U6 selected from the reactions (Supplementary Figure S10). Unpaired *t*-test with Welch's correction of the three replicates (*P* = 0.8774). (**E**) Cwc2-U6 crosslinks in the presence of wild-type Prp16 or *prp16-302*. Yeast whole cell extract was made from yeast strains containing TAP-tagged Cwc2. Splicing reactions with *ACT1* pre-mRNA were UV irradiated before selection with a biotinylated oligonucleotide complementary to U6 snRNA, under denaturing conditions. elected proteins were separated by SDS-PAGE and Cwc2-TAP was detected with anti-TAP antibody by immunoblotting. Levels of Cwc2 crosslinked to U6 were quantified in three separate experiments and normalized to the amount of U6 selected from the reactions (Supplementary Figure S12). Unpaired *t*-test with Welch's correction of the three replicates (*P* = 0.0046). (**F**) Cwc2-pre-mRNA crosslinks in the presence of wild-type Prp16 or *prp16-302*. Yeast whole cell extract was made from yeast strains containing TAP-tagged Cwc2. Splicing reactions with biotin-containing *ACT1* pre-mRNA were UV irradiated before selection with streptavidin paramagnetic beads, under denaturing conditions. Selected proteins were separated by SDS-PAGE and Cwc2-TAP was detected with anti-TAP antibody by immunoblotting. Levels of Cwc2 crosslinked to pre-mRNA were quantified in three separate experiments and normalized to the amount of U6 selected from the reactions (Supplementary Figure S13). Unpaired *t*-test with Welch's correction of the three replicates (*P* = 0.0037).

The functional significance of Cwc2 crosslinking to the U6 snRNA and the pre-mRNA is not clear (29,40). Our data showing a functional link between Cwc2 and Prp16 indicate that Cwc2, which stabilizes the U2-U6 helix I, may be the target for Prp16 destabilization of U2-U6 helix I before the second step of splicing. If Cwc2 is a target of Prp16, then mutation in Prp16 may influence the interaction of Cwc2 with the U6 snRNA and/or the pre-mRNA. To test whether Prp16 mutation influenced Cwc2 interaction with the U6 snRNA, we added either wild-type Prp16 or the dominant negative Prp16 D473A mutant to splicing extracts made from a strain containing TAP-tagged Cwc2. The Prp16 D473A protein blocks the second step of splicing when added to yeast whole cell splicing extracts (Supplementary Figure S6). Splicing was initiated with *ACT1* pre-mRNA, then reactions were UV crosslinked and a biotinylated oligonucleotide complementary to the U6 snRNA was used to select out the U6 snRNA and any associated proteins using streptavidin-coated magnetic beads under stringent conditions (0.5 M NaCl and 0.5% SDS, see the Materials and Methods section). Crosslinking of Cwc2-TAP to the U6 snRNA was quantitated in three independent reactions by immunoblotting with anti-TAP antibodies and values normalized to the amount of U6 snRNA selected from each reaction (Figure 7A and Supplementary Figure S7). As a control, identical splicing reactions were subjected to selection of U6 under the same denaturing conditions without UV irradiation and we observed no crosslinking of Cwc2 to U6 (Supplementary Figure S7). Comparing the amount of Cwc2 crosslinked to U6 in the presence of wild-type Prp16 and Prp16 D473A, we observed stabilization of Cwc2 binding to the U6 snRNA in the presence of Prp16 D473A (Figure 7A). Statistical analysis of these data revealed that the differences were not statistically significant. However, these data still may have functional relevance (see the Discussion section). Next we tested whether the Prp16 D473A mutation influenced Cwc2 crosslinks to the pre-mRNA. In this case, biotinylated *ACT1* pre-RNA was used to initiate splicing in Cwc2-TAP extracts followed by UV irradiation to induce Cwc2 crosslinking to the pre-mRNA. Streptavidin-coated magnetic beads were used to select the pre-mRNA from the reactions under stringent conditions. Crosslinking of Cwc2 to the pre-mRNA was quantitated in three independent reactions by immunoblotting with anti-TAP antibodies and values normalized to the amount of pre-mRNA selected from each reaction (Figure 7B and Supplementary Figure S8). As a control identical splicing reactions were subjected to selection of pre-mRNA under the same denaturing conditions without UV irradiation and we observed no crosslinking of Cwc2 to pre-mRNA (Supplementary Figure S8). Comparing the amount of Cwc2 crosslinked to the pre-mRNA in the presence of wild-type Prp16 and Prp16 D473A we observed stabilization of Cwc2 binding to the pre-mRNA in the presence of Prp16 D473A (Figure 7B). Again, statistical analysis of these data revealed that the differences were not statistically significant. However, these data may still have functional relevance (see the Discussion section).

To determine the influence of Prp2 on the interactions of Cwc2 with the U6 snRNA and the pre-mRNA, we repeated the experiments shown in Figure 7A and B with a dominant negative mutation in the ATPase Prp2. The dominant negative Prp2-LAT mutant, which contains a serine to leucine mutation in the conserved ATPase domain SAT motif III, blocks the first step of splicing when added to yeast whole cell splicing extracts (Figure S6). Wild-type Prp2 or Prp2-LAT were added to Cwc2-TAP splicing extract and crosslinking of Cwc2 to U6 or the pre-mRNA was quantitated in three independent reactions and normalized to the amount of U6 or pre-mRNA selected from each reaction (Figure 7C and D, Supplementary Figures S9 and S10). As a control, identical splicing reactions were subjected to selection of U6 and pre-mRNA under the same denaturing conditions without UV irradiation and we observed no crosslinking of Cwc2 to U6 or the pre-mRNA (Supplementary Figures S9 and S10). When comparing splicing reactions in the presence of wild-type Prp2 and Prp2-LAT, we observed no change in the intensity of crosslinking of Cwc2 to the U6 snRNA (Figure 7C). In contrast, comparing the amount of Cwc2 crosslinked to the pre-mRNA in the presence of wild-type Prp2 and Prp2-LAT we observed stabilization of Cwc2 binding to the pre-mRNA in the presence of Prp2-LAT (Figure 7D). Statistical analysis of these data revealed that the differences were not statistically significant. However, these data may still have functional relevance (see the Discussion section).

As the *prp16-302* mutation was suppressed by mutation in Cwc2, we decided to investigate what influence *prp16-302* had on Cwc2 crosslinking to U6 and the pre-mRNA. Yeast strains containing Cwc2-TAP were constructed where the genomic copy of *PRP16* was deleted and Prp16 function complemented by a plasmid with either wild-type Prp16 or *prp16-302*. Extracts from these strains were used in splicing reactions with *ACT1* pre-mRNA, with both extracts assembling spliceosomes equally well (Supplementary Figure S11). The splicing reactions were then subjected to UV irradiation and crosslinking of Cwc2 to the U6 snRNA or pre-mRNA was quantitated in three independent reactions by immunoblotting with anti-TAP antibodies and values normalized to the amount of pre-mRNA selected from each reaction (Figure 7E and F, Supplementary Figures S12 and S13). As a control, identical splicing reactions were subjected to selection of U6 and pre-mRNA under the same denaturing conditions without UV irradiation and we observed no crosslinking of Cwc2 to U6 or the pre-mRNA (Supplementary Figures S12 and S13). We found a statistically significant increase in the crosslinking of Cwc2 to the U6 snRNA in the presence of the *prp16-302* mutation (Figure 7E). This increased interaction, or stabilization, of Cwc2 with the U6 snRNA further supports the idea that Cwc2 stabilizes interactions of the U6 snRNA and that Prp16 targets Cwc2 for destabilization. We also found a significant decrease in the crosslinking of Cwc2 to the pre-mRNA in the presence of the *prp16-302* mutation (Figure 7F). This destabilization of Cwc2 with the pre-mRNA in the presence of the *prp16-302* mutation suggests that the action of Prp16 stabilizes the interaction of Cwc2 with the pre-mRNA.

## DISCUSSION

The DExD/H-box ATPases are required to remodel the spliceosome for progression through the two steps of splicing, and for kinetic proofreading of these remodeling steps. Prp2 action is required before the first step of splicing to remodel the spliceosome for the first step (10,11). The ATPase Prp16 proofreads the first step of splicing and remodels the spliceosome for progression between the first and second steps of splicing (22–24). We show here that the NTC protein Cwc2 is one target of Prp2 and Prp16 action. Cwc2 functionally interacts with the U6 snRNA ACAGAGA sequence, the U6 ISL and the U2-U6 helix I, all RNA elements proposed to contribute to the active site of the spliceosome. We found that one function of Cwc2 is to stabilize the U2-U6 helix I that antagonizes the destabilization of U2-U6 helix I by Prp16 between the two steps of splicing. Indeed, mutation of Prp16 results in the stabilization of Cwc2 with the U6 snRNA indicating that Cwc2 is a target of Prp16 remodeling of the spliceosome between the two steps of splicing.

### Possible function of the Cwc2 Torus domain α helix 1

Two regions of Cwc2 were identified as being functionally important for genetic interactions with the U2 and U6 snRNAs. These regions of Cwc2 were the RRM (F183) and α helix 1 (Y34 and W37) of the Torus domain. The Cwc2 Torus domain α helix 1 contains a number of highly conserved aromatic residues that form an interface with the Zn finger domain, allowing the Torus domain to enclose the Zn finger (31). Structural comparison of the Cwc2 Zn finger with the closely related TIS11D Zn finger bound to RNA revealed that interaction of the Torus domain near α helix 1 would need to be disrupted to allow RNA recognition similar to that seen for TIS11D (31,41). The mutations identified here in Cwc2, Y34A and W37A, could disrupt the interaction of the Torus domain α helix 1 with the Zn finger and allow access to RNA. As the Y34A and W37A mutations displayed synthetic growth defects with U6 mutations in the ACAGAGA and helix I regions, it appears that the α helix 1 interface with the Zn finger is required for Cwc2 functional interaction with the U6 ACAGAGA and helix I. However, disruption of the Torus domain interface with the Zn finger could still be required at some stage during splicing to allow RNA access to the Zn finger. Interestingly, no genetic interactions were found between the Torus domain α helix 1 mutants Y34A and W37A and the U6 ISL. Genetic interactions with the U6 ISL were limited to the RRM. Our functional genetic results complement previous crosslinking experiments that found the RRM interacts with the U6 ISL and other regions of the U6 snRNA whereas the Zn finger, connector and Torus domains did not interact with the U6 ISL (31).

Another interesting observation was the dichotomy of *in vivo* splicing phenotypes found with the Cwc2 mutations using the *ACT1-CUP1* reporters. The two Torus domain α helix 1 mutations Y34A and W37A only decreased the efficiency of the first step of splicing indicating a role in maintaining a conformation required for the first step of splicing. In contrast, only the RRM mutation F183D reduced fidelity of 3' splice site recognition indicating a role in the Prp16-dependent progression to the second step of splicing. This difference in splicing phenotype between mutants indicates that the Torus domain and RRM have different functions. As we have shown that only the RRM interacts genetically with the U6 ISL and others have shown that only the RRM interacts with the U6 ISL by crosslinking (31) it appears that the Cwc2 RRM interaction with the U6 ISL is required for fidelity of 3' splice site recognition.

### Prp16 targets Cwc2 during splicing

Remodeling of the spliceosome involves changes in protein composition/interactions, protein–RNA interactions as well as changes in RNA–RNA interactions. The NTC is present at the complex B stage of the splicing cycle and includes Isy1 (16). Transition from the B complex to the B^act^ complex, which is competent for the first step of splicing, involves removal of the U1 and U4 snRNAs and approximately 35 associated proteins (16). At this stage, the B^act^ complex gains 12 proteins, 10 of which are additional NTC related proteins including Cwc2 (16). Cwc2 and Isy1 appear to have a related function as we have shown here that the *in vivo* splicing phenotype of Cwc2 mutants, decreasing the first step of splicing and reducing fidelity of 3' splice site recognition, is the same as that found by Isy1 deletion (35). In addition, we find that deletion of Isy1 prevents the growth of a Cwc2 deletion strain at 25°C and 16°C indicating that Cwc2 and Isy1 functionally cooperate. Combining Cwc2 and Ecm2 deletions, on the other hand, increases growth slightly, suggesting that Ecm2 may antagonize Cwc2 function. Cwc2 and Isy1 have been shown to interact by the two-hybrid assay (42). Interestingly, Villa and Guthrie have proposed that during the transition between the two steps of splicing Prp16 remodels an interaction involving Isy1, but that Isy1 is not the only target of Prp16 activity (35). We propose that Cwc2 and Isy1 together stabilize a spliceosome conformation that is then remodelled by Prp16 during the transition between the two steps of splicing.

Through analysis of the Cwc2 crystal structure, it has been proposed that the connector element of Cwc2, a flexible and conserved region of Cwc2 that connects the Torus and RRM domains, may be a molecular switch that modulates RNA contacts of Cwc2 (31). Binding of a protein ligand to this connector region has been proposed to either promote or prevent certain RNA interactions of Cwc2 (31). We propose that Prp2 and Prp16 may be the protein ligands that interact either directly with Cwc2, or indirectly through other proteins, to modulate the RNA contacts of Cwc2 during spliceosome remodeling. We have found that mutation in Prp2 may influence interactions of Cwc2 with the pre-mRNA and mutation in Prp16 influences the interactions of Cwc2 with both the pre-mRNA and the U6 snRNA.

Consistent with the role of Cwc2 in stabilizing U2-U6 helix I and Prp16 in destabilizing U2-U6 helix I, the *prp16-302* mutation results in the stabilization of Cwc2 interactions with U6 and destabilization of Cwc2 interactions with the pre-mRNA, indicating a role for Prp16 in destabilization of Cwc2 interaction with U6 and stabilization of Cwc2 interaction with the pre-mRNA. In addition, the dominant negative mutation Prp16 D473A also appears to stabilize Cwc2 interaction with U6 and the pre-mRNA before the second step of splicing. While a trend is observed, the stabilization of Cwc2 interaction with U6 and the pre-mRNA by dominant negative Prp16 D473A is quite subtle and does not show a statistical significance. However, lack of statistical significance does not necessarily mean that the changes observed have no functional relevance. Recent analysis using a purified yeast splicing system to investigate the second step of splicing has also found that Prp16-mediated remodeling is subtle and may only involve remodeling of interactions at the catalytic core of the spliceosome (43). Therefore, even though the trends we observe in Cwc2 association with the U6 snRNA and pre-mRNA, in some cases, do not display statistical significance, they may still have functional relevance. Furthermore, the replicates that do not appear to conform as well to the trends observed (Figure 7B, replicate 1 and Figure 7D, replicate 2) may be influenced by our normalization to primer extension values for selected pre-mRNA (see Supplementary Figure S8C, lanes 2 versus 4 and Supplementary Figure S10C, lanes 6 versus 8) where the primer extension does not appear to have worked as well as expected. It remains to be seen whether the subtle changes we observed with the Prp16 D473A on Cwc2 interactions suggests a role in Prp16 destabilizing Cwc2/U6 and Cwc2/pre-mRNA interactions before the second step of splicing. In addition, it is still not clear whether the subtle changes of Cwc2 interaction with the pre-mRNA induced by Prp2 mutation indicate a function for Prp2 in destabilizing Cwc2/pre-mRNA interactions before the first step of splicing. Importantly, our data do provide statistically proven evidence that Prp16 remodeling results in destabilization of Cwc2 interaction with the U6 snRNA and stabilization of Cwc2 interaction with the pre-mRNA.

### Cwc2 and RNA structures required for splicing

The evidence presented here suggests that the cause of the synthetic lethality between Cwc2 Y34A, Cwc2 F183D and U6-A56C,U57C is due to the destabilization of U2-U6 helix I. It was surprising, then, that the U2-A27C,U28C mutation, which disrupts base-pairing between the U6-A56 and U6-U57 nucleotides in helix I, did not display genetic interaction with Cwc2. U6 mutations in U2-U6 helix I are much more severe than mutations in U2, suggesting that the U6 nucleotides have other functions in addition to base-pairing (44–46). However, repairing the base-pairing with the complementary nucleotides in U2 suppressed the synthetic lethality between the Cwc2 alleles and the U6-A56C,U57C mutation. One way of interpreting these data is that the U6-A56 U6-U57 nucleotides must be involved in another interaction with either RNA or protein that is functionally redundant with the base-pairing of U2-U6 helix I, and that these interactions do not involve the U2 nucleotides U2-A27 U2-U28. It is likely that Cwc2 is functionally redundant with a process in splicing that requires the integrity of U2-U6 helix I, and the dual function of the U6-A56 U6-U57 nucleotides, but not the U2-A27 U2-U28 nucleotides. Hence, for splicing to occur there must be at least two of these three features intact: the U6-A56 U6-U57 nucleotides; the base-pairing of U2-U6 helix I (or stabilization through cold temperature); a functioning Cwc2. With two of these features eliminated, splicing is inhibited, most likely at the first step.

Extending helix Ia with the U6-A53U substitution displays synthetic sickness with the Cwc2 W37A allele, which is exacerbated by lower temperatures. Changing U6-A53 to C or G is viable at all temperatures, however A53U is temperature sensitive at 37°C, indicating that the base-pairing potential of helix I may be a cause of the temperature sensitive phenotype (47). The genetic interactions presented in this study support a role where Cwc2 stabilizes a conformation including helix I. The U6-A53U allele extends helix I and is, therefore, expected to stabilize the structure. The synthetic lethal interaction of Cwc2 W37A with U6-A53U, and Cwc2 F183D with U6-Ins 1U A53 is more severe at the lower temperatures indicating that the two mutations together are causing a conformation to become hyper-stabilized, resulting in inhibition of splicing at lower temperature. Taken together, these data would indicate that Cwc2 functions to maintain U2-U6 helix I at a specific point in the splicing cycle, however helix I must remain dynamic enough to remodel between the two steps, as mutations which increase base-pairing potential cause hyper-stabilization and are synthetic lethal/sick with Cwc2 mutations at lower temperatures, whereas mutations which destabilize helix I are synthetic lethal with Cwc2 at higher temperatures. Cwc2 also displays genetic interaction with two mutations that destabilize helix Ib. The Cwc2 W37A mutation is synthetic sick with the U6-A59C mutation, and this phenotype is exacerbated at lower temperatures, with no growth observed at 16°C. The Cwc2 F183D mutation is synthetically lethal at 37°C with A and G substitutions at U2-C22. The A and G substitutions at U2-C22 also destabilize U2-U6 helix Ib as the synthetic lethality between Cwc2 F183D and substitutions at U2-C22 are only present at 37°C and not at lower temperatures tested. Therefore, destabilization of helix Ib is the likely cause of the synthetic lethality between Cwc2 F183D and substitutions at U2-C22.

The U6–A59C mutation, located in the AGC triad, results in decreased exon ligation both *in vitro* and *in vivo* (44,48). U6-A59C is temperature sensitive at 37°C, and this phenotype can be suppressed by repairing the base-pairing in helix Ib. Interaction between Cwc2 and U6-A59C is more severe at lower temperatures, as opposed to the interactions found between Cwc2 and the U6 helix Ia mutant U6-A56C,U57C where synthetic lethal interactions were not present at lower temperatures. This would imply that a conformation of the spliceosome has become hyper-stable in the presence of the two mutations, and at lower temperatures splicing is blocked. Evidence from the structure of a group II self-splicing intron suggests that the AGC triad functions in tertiary interactions with the U6 ISL and ACAGAGA sequence, which has recently been supported in the spliceosome (4,49). It is possible that Cwc2 may help mediate these tertiary interactions.

We have shown here that Cwc2 interacts genetically with the U6 ACAGAGA, the U6 ISL and the U2-U6 helix I suggesting a functional link to these regions of U6 that are thought to contribute to the active site of the spliceosome. The U6 ACAGAGA, the U6 ISL and the U2-U6 helix I regions have been proposed to interact to form a putative catalytic triple helix in the spliceosome similar to that found in the self-splicing group II introns (3). Cwc2 has been hypothesized to directly, or indirectly, promote the putative catalytic triple helix conformation of the spliceosome and to promote an active conformation of the U6 ISL (32). We have found that Cwc2 can at least stabilize the first step conformation of the spliceosome by stabilizing U2-U6 helix I. However, the functional genetic interactions of Cwc2 with the U6 ACAGAGA, the U6 ISL and the U2-U6 helix I support the hypothesis that Cwc2 stabilizes other RNA conformations like the putative catalytic triple helix.

## SUPPLEMENTARY DATA

Supplementary Data are available at NAR Online.

SUPPLEMENTARY DATA
