# Supplementary material for: Remodeling of U2-U6 snRNA helix I during pre-mRNA splicing by Prp16 and the NineTeen Complex protein Cwc2
Source: Nucleic Acids Res. 2014 May 21;42(12):8008–23. doi: 10.1093/nar/gku431 (PMC4081067; doi:10.1093/nar/gku431)
Supplement: SUPPLEMENTARY DATA [file supp_42_12_8008__index.html]

Remodeling of U2-U6 snRNA helix I during pre-mRNA splicing by Prp16 and the NineTeen Complex protein Cwc2 — SUPPLEMENTARY DATA 

# Remodeling of U2-U6 snRNA helix I during pre-mRNA splicing by Prp16 and the NineTeen Complex protein Cwc2

## SUPPLEMENTARY DATA

**Files in this Data Supplement:**

- SUPPLEMENTARY DATA
