# Supplementary material for: Remodeling of U2-U6 snRNA helix I during pre-mRNA splicing by Prp16 and the NineTeen Complex protein Cwc2
Source: Nucleic Acids Res. 2014 May 21;42(12):8008–23. doi: 10.1093/nar/gku431 (PMC4081067; doi:10.1093/nar/gku431)
Supplement: SUPPLEMENTARY DATA [file supp_gku431_nar-02137-a-2013-File009.pdf]

SUPPLEMENTARY DATA FOR

Remodelling of U2-U6 snRNA helix I during pre-mRNA splicing by Prp16 and the nineteen complex protein Cwc2

Rebecca Hogg, Rogerio Alves de Almeida, Jayalath P.D. Ruckshanthi and Raymond T. O'Keefe

SUPPLEMENTARY TABLE 1

SUPPLEMENTARY FIGURES S1-13

SUPPLEMENTARY REFERENCES

Supplementary Table 1 Yeast strains

Strain	Genotype	Source
Y23907<br>BY4743	MAT a/α; his3Δ1/his3Δ; leu2Δ0/leu2Δ0; lys2Δ0/LYS2; MET15/met15Δ0; ura3Δ0/ura3Δ0; YDL209c::kanMX4/YDL209c	EUROSCARF
CWC2/U2 KO diploid	MAT a/α; his3Δ1/his3Δ; leu2Δ0/leu2Δ0; lys2Δ0/LYS2; MET15/met15Δ0; ura3Δ0/ura3Δ0; YDL209c::kanMX4/YDL209c; SNR20::hphNT1/SNR20	This study
CWC2/U4 KO diploid	MAT a/α; his3Δ1/his3Δ; leu2Δ0/leu2Δ0; lys2Δ0/LYS2; MET15/met15Δ0; ura3Δ0/ura3Δ0; YDL209c::kanMX4/YDL209c; SNR14::hphNT1/SNR14	This study
CWC2/U5 KO diploid	MAT a/α; his3Δ1/his3Δ; leu2Δ0/leu2Δ0; lys2Δ0/LYS2; MET15/met15Δ0; ura3Δ0/ura3Δ0; YDL209c::kanMX4/YDL209c; SNR7::hphNT1/SNR7	This study
CWC2/U6 KO diploid	MAT a/α; his3Δ1/his3Δ; leu2Δ0/leu2Δ0; lys2Δ0/LYS2; MET15/met15Δ0; ura3Δ0/ura3Δ0; YDL209c::kanMX4/YDL209c; SNR6::hphNT1/SNR6	This study
YCWC2KO	MATα; his3Δ; leu2Δ0; lys2Δ0; ura3Δ0; YDL209c::kanMX4; pRS416-CWC2	McGrail et. al. (2009)
CWC2KO/U2KO	MATα; his3Δ; leu2Δ0; LYS2; MET15; ura3Δ0; YDL209c::kanMX4; SNR20::hphNT1; pRS416-CWC2-U2	This study
CWC2KO/U4KO	MATα; his3Δ; leu2Δ0; LYS2; MET15; ura3Δ0; YDL209c::kanMX4; SNR14::hphNT1; pRS416-CWC2-U4	This study
CWC2KO/U5KO	MATα; his3Δ; leu2Δ0; LYS2; MET15; ura3Δ0; YDL209c::kanMX4; SNR7::hphNT1; pRS416-CWC2-U5	This study
CWC2KO/U6KO	MATα; his3Δ; leu2Δ0; LYS2; MET15; ura3Δ0; YDL209c::kanMX4; SNR6::hphNT1; pRS416-CWC2-U6	This study
CWC2/PRP16KO diploid	MAT a/α; his3Δ1/his3Δ; leu2Δ0/leu2Δ0; lys2Δ0/LYS2; MET15/met15Δ0; ura3Δ0/ura3Δ0; YDL209c::kanMX4/YDL209c; YKR086W::hphNT1/YKR086W	This study
CWC2/PRP16KO	MAT a; his3Δ; leu2Δ0; LYS2; met15Δ0; ura3Δ0; YDL209c::kanMX4; YKR086W::hphNT1; pRS416-Cwc2-Prp16	This study
CWC2/PRP22KO haploid	MAT α; his3Δ; leu2Δ0; ura3Δ0; MET15; YDL209c::kanMX4; YER013W::hphNT1; pRS416-Cwc2-Prp22	This study
YCL51	MATa; cup1Δ::ura3; leu2; ura3; trp1; lys2; ade; his3; GAL+	Provided by Jean Beggs

CWC2KO/CUPKO	MATa; leu2; ura3; trp1; lys2; his3; met15Δ0; GAL+; cup1Δ::ura3; YDL209c::kanMX4; pRS416-CWC2	This study
CWC2 ISY1 KO	MATα; his3Δ; leu2Δ0; lys2Δ0; ura3Δ0; YDL209c::kanMX4; YJR050w::hphNT1; pRS416-CWC2	This study
CWC2 ECM2 KO	MATα; his3Δ; leu2Δ0; lys2Δ0; ura3Δ0; YDL209c::kanMX4; YBR065c::hphNT1; pRS416-CWC2	This study
YCWC2TAP	Mat a; prc1-407; prb1-122; pep4-3; leu2; trp1; ura3-52; gal2; YDL209C-TAP	McGrail et. al. (2009)
YCWC2TAP-PRP16	Mat a; prc1-407; prb1-122; pep4-3; leu2; trp1; ura3-52; gal2; YDL209C-TAP; YKR086W::hphNT1; pRS416-PRP16	This study
YCWC2TAP-*PRP16-302*	Mat a; prc1-407; prb1-122; pep4-3; leu2; trp1; ura3-52; gal2; YDL209C-TAP; YKR086W::hphNT1; pRS416-*prp16-302*	This study

A

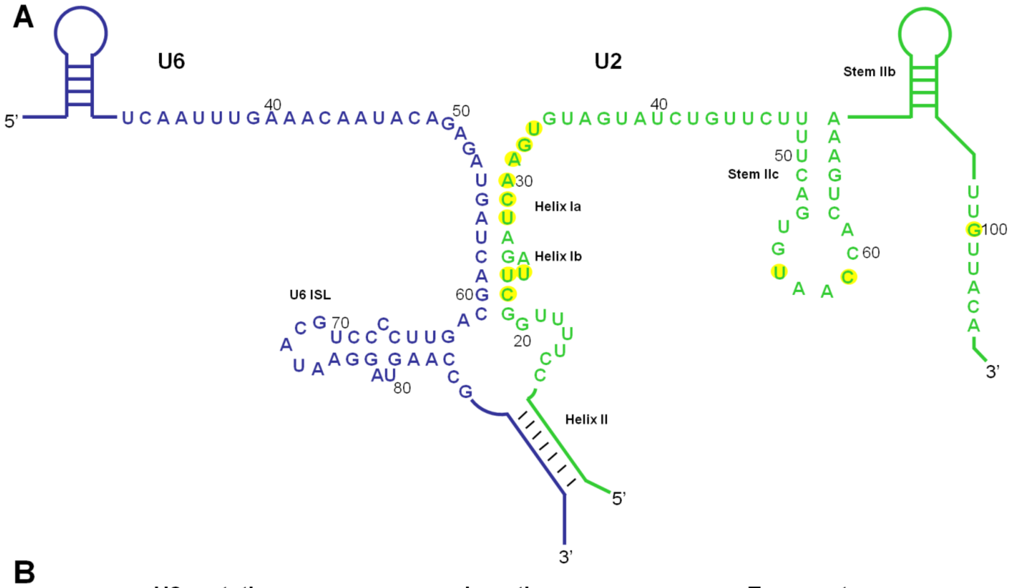

B

U2 mutation	Location	Temperature sensitivity
C22A	Helix Ib	39°C
C22G	Helix Ib	39°C
U23C	Helix Ib	39°C
U23G	Helix Ib	39°C
U24A	Bulge between helix Ia and Ib	-
U24C	Bulge between helix Ia and Ib	-
U24G	Bulge between helix Ia and Ib	-
ΔU24	Bulge between helix Ia and Ib	-
A25C	Bulge between helix Ia and Ib	-
A25G	Bulge between helix Ia and Ib	-
A25U	Bulge between helix Ia and Ib	-
ΔA25	Bulge between helix Ia and Ib	-
A27C,U28C	Helix Ia	37°C
Ins 1U U28/A29	Helix Ia	39°C
Ins 1U A30/A31	Helix Ia	39°C
Ins 1U G32/U33	Helix Ia	-
U56C	U2 stem IIc	-
C59G	U2 stem IIc	-
C59U	U2 stem IIc	-
G100A	U2 stem IIc	-

Figure S1. U2 alleles used in to investigate genetic interactions with Cwc2

(A) Secondary structure diagram of the interactions between U2 and U6 snRNAs required before the first step of splicing, showing U2 stem IIc. Location of U2 mutations are noted by a yellow circle around the nucleotide. (B) Mutations used in the plasmid shuffle assay with Cwc2. Mutant U2 and *cwc2* alleles were used as the sole source of U2 snRNA and Cwc2 to reveal any genetic interaction between Cwc2 and U2. Temperature sensitive phenotypes are noted. – indicates no temperature sensitivity.

A

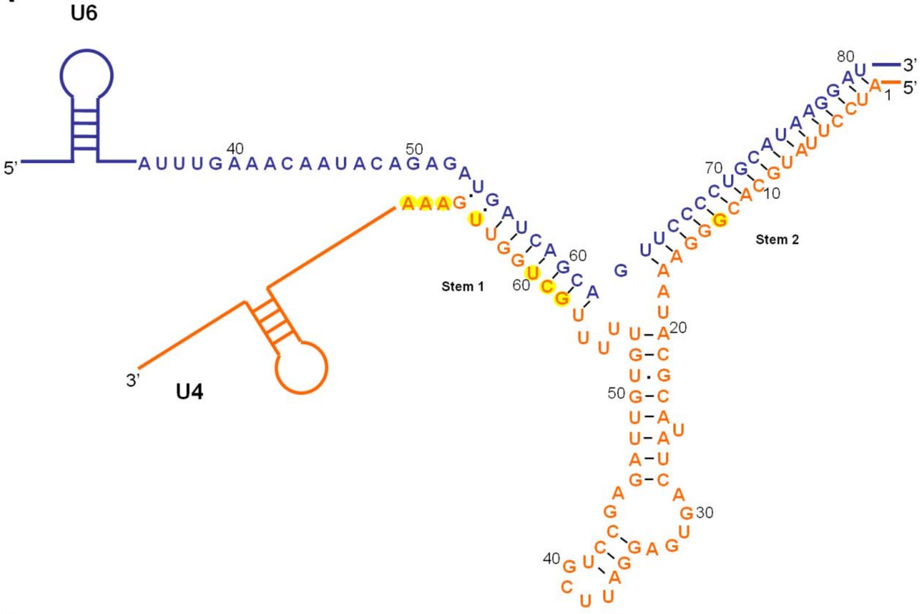

B

U4 mutation	Location	Temperature sensitivity
G14C	Stem II	23°C
G58A	Stem I	37°C
C59U	Stem I	39°C
U60C	Stem I	-
U64G	Stem I	-
A66U,A67U,A68G (cs-1)	Stem I	23°C

Figure S2. U4 alleles used in to investigate genetic interactions with Cwc2

(A) Secondary structure of the RNA-RNA interactions within the U4/U6 di-snRNP. Locations of U4 mutations are noted by a yellow circle around the nucleotide. (B) Mutations used in the plasmid shuffle assay with *cwc2* alleles. Mutant U4 and *cwc2* alleles were used as the sole source of U4 snRNA and Cwc2 to reveal any genetic interaction between Cwc2 and U4. The *cs-1* mutation blocks U4 dissociation during spliceosome assembly by increasing the base pairing between U4 and U6 (Li and Brow 1996; Kuhn et al. 1999). Temperature sensitive phenotypes are noted. – indicates no temperature sensitivity.

A

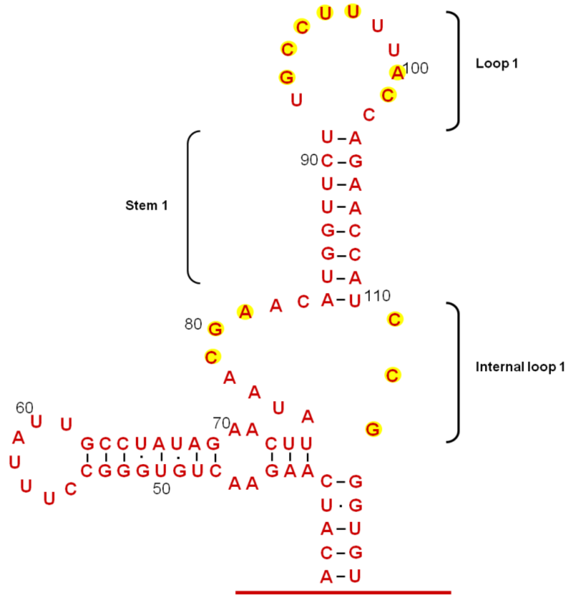

B

U5 mutation	Location	Temperature sensitivity
ΔC79,G80	Internal loop 1	37°C
ΔC79-A81	Internal loop 1	-
ΔC111	Internal loop 1	-
ΔC112,G113	Internal loop 1	37°C
U5 short form (Δ179-214)		-
A100U,C101G	Loop 1	-
ΔG93	Loop 1	-
ΔC94,C95	Loop 1	37°C
ΔU96,U97	Loop 1	37°C
ΔC101	Loop 1	39°C

Figure S3. U5 alleles used in to investigate genetic interactions with Cwc2

(A) Secondary structure of the U5 snRNA showing nucleotides 36-118. Locations of U5 mutations are noted by a yellow circle around the nucleotide. (B) U5 mutations used in the plasmid shuffle assay with the *cwc2* alleles. Mutant U5 and *cwc2* alleles were used as the sole source of U5 snRNA and Cwc2 to reveal any genetic interaction between Cwc2 and U5. The U5 short form was also used in the assay. The U5 snRNA exists as two forms in yeast, with a short form lacking the 3' terminal stem loop (Patterson and Guthrie, 1987). Temperature sensitive phenotypes are noted. – indicates no temperature sensitivity.

A

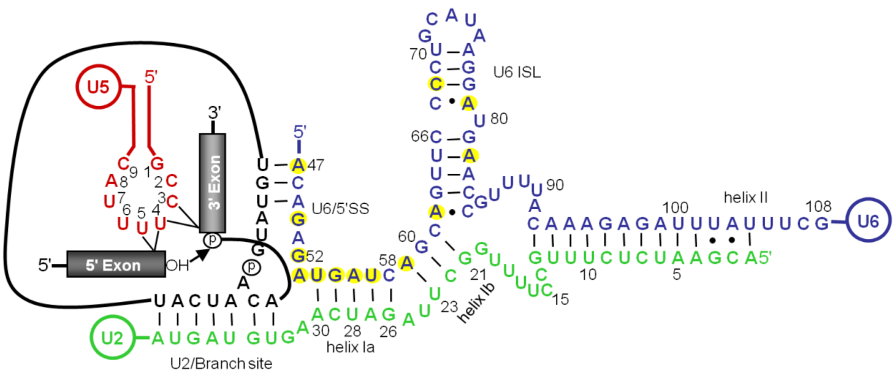

B

U6 mutation	Location	Temperature sensitivity
A47G	5' splice site to Helix Ia	37°C
G50U	5' splice site to Helix Ia	37°C
G52U	5' splice site to Helix Ia	37°C
A53U	5' splice site to Helix Ia	37°C
Ins 1U A53/U54	5' splice site to Helix Ia	37°C
U54C	Helix Ia	-
G55A	Helix Ia	-
U54C,G55A	Helix Ia	-
Ins 1U G55/A56	Helix Ia	37°C
U57A	Helix Ia	-
U57C	Helix Ia	-
A56C,U57C	Helix Ia	37°C
C58U	Helix Ia	37°C
A59C	Helix Ib	37°C
A62G	ISL	16°C
A79G	ISL	16°C
C68U	ISL	-
A82G	ISL	-

Figure S4. U6 alleles used in to investigate genetic interactions with Cwc2

(A) Diagram of RNA interactions within the catalytic core of the spliceosome before the second step of splicing. Location of U6 mutations are noted by a yellow circles around the nucleotide. (B) U6 mutations used in the plasmid shuffle assay with the *cwc2* alleles. Mutant U6 and *cwc2* alleles were used as the sole source of U6 snRNA and Cwc2 to reveal any genetic interaction between Cwc2 and U6. Temperature sensitive phenotypes are noted. – indicates no temperature sensitivity.

A

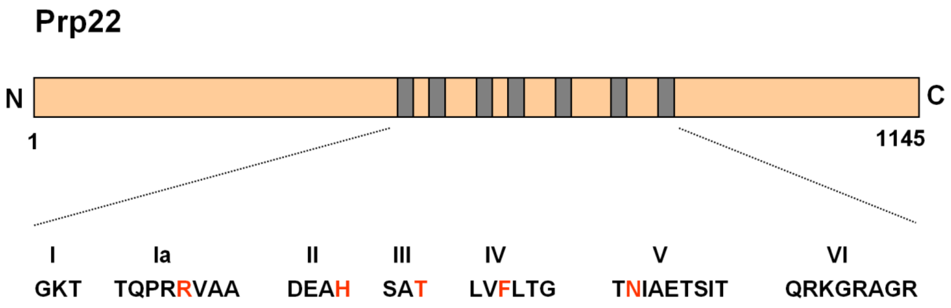

B

Prp22 mutation	Location	Temperature sensitivity
R538A	Domain Ia	16°C
H606A	Domain II	20°C
T637A	Domain III	25°C
F697A	Domain IV	30°C
N758A	Domain V	16°C

Figure S5. *prp22* alleles used to investigate genetic interactions between Prp22 and Cwc2

(A) Schematic diagram of Prp22 showing the seven conserved motifs by which DExD/H box proteins are defined. Sequences I-VI are that of Prp22. Mutated residues which were substituted with alanine are noted in red. (B) Prp22 mutations, and their location, used in the plasmid shuffle assay with the Cwc2 mutants. Mutant Prp22 and *cwc2* alleles were used as the sole source of Prp22 and Cwc2 proteins to reveal any genetic interaction between Cwc2 and Prp22. Temperature sensitive phenotypes are noted (Campodonico and Schwer, 2002; Schneider et al., 2004)

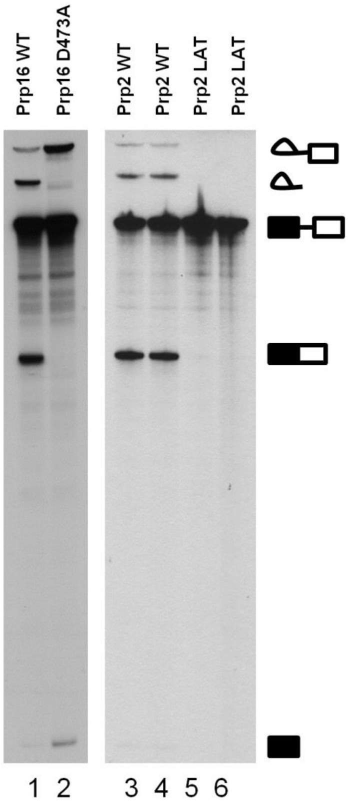

Figure S6. Influence of dominant negative Prp16 D473A and Prp2 LAT proteins on *in vitro* pre-mRNA splicing

Yeast whole cell extract was incubated with wild-type Prp16 (lane 1), Prp16 D473A (lane 2), wild-type Prp2 (lane 3 and 4) or Prp2 LAT (lane 5 and 6) for 15 min. at 23°C. Radioactively labelled *ACT1* pre-mRNA was added and the reactions incubated a further 20 min. RNA from each reaction was isolated and then separated on a denaturing polyacrylamide gel. The gels were then fixed and dried then exposed to x-ray film to detect the *ACT1* pre-mRNA, splicing intermediates and splicing products which are schematically represented on the right hand side of the figure.

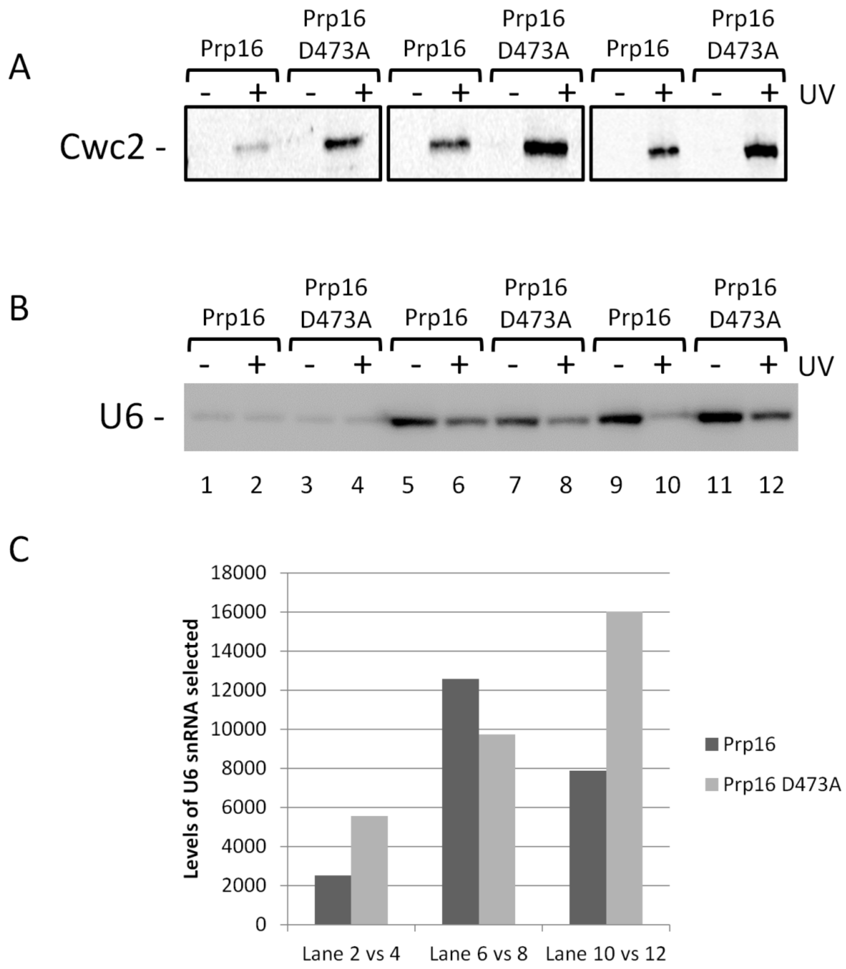

Figure S7. Western blotting and primer extension of Cwc2 and U6 snRNA selected from crosslinked splicing reactions in the presence of Prp16 and Prp16 D473A

(A) Western blotting of Cwc2 crosslinked to the U6 snRNA from three replicate splicing reactions. (B) Primer extension analysis of U6 snRNA selected from three replicate splicing reactions. (C) Levels of U6 snRNA selected in reactions subjected to UV irradiation in Prp16 vs Prp16 D473A supplemented extracts were quantified by phosphorimaging.

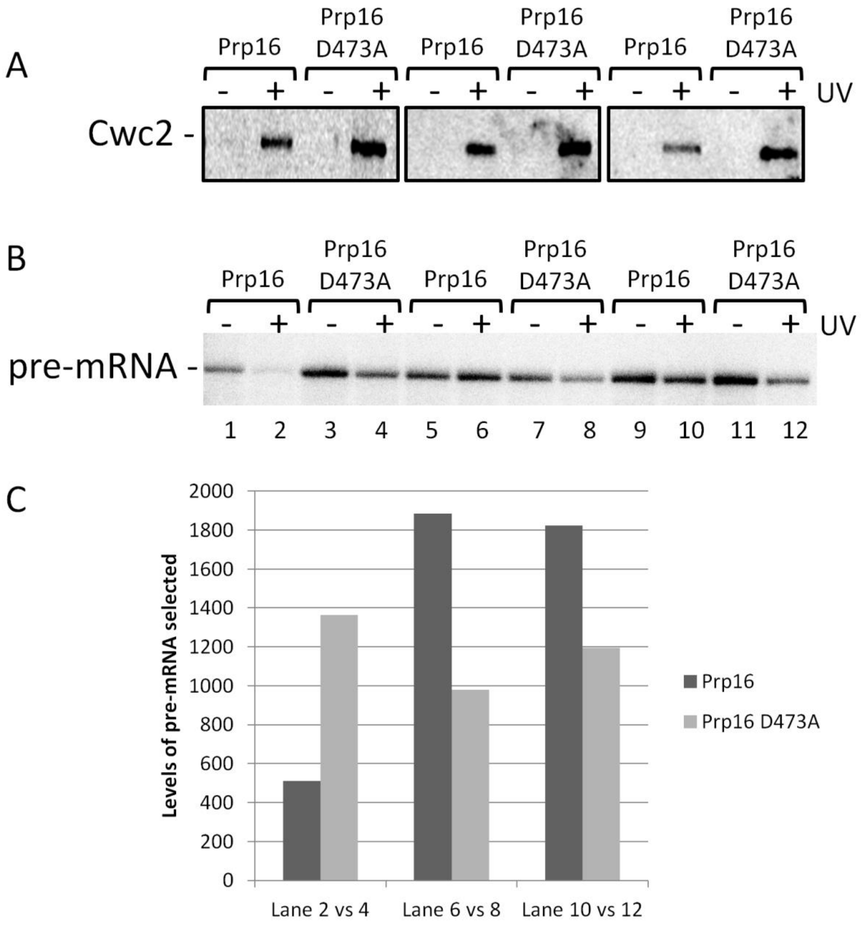

Figure S8. Western blotting and primer extension of Cwc2 and pre-mRNA selected from crosslinked splicing reactions in the presence of Prp16 and Prp16 D473A

(A) Western blotting of Cwc2 crosslinked to the pre-mRNA from three replicate splicing reactions. (B) Primer extension analysis of pre-mRNA selected from three replicate splicing reactions. (C) Levels of pre-mRNA selected in reactions subjected to UV irradiation in Prp16 vs Prp16 D473A supplemented extracts were quantified by phosphorimaging.

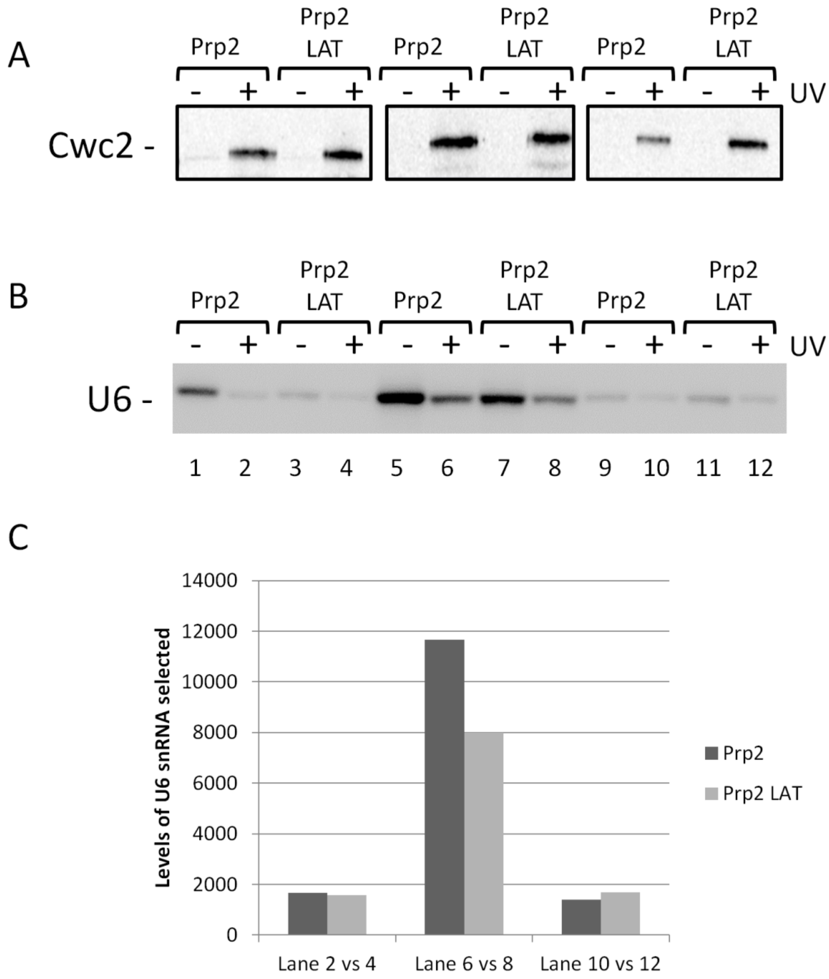

Figure S9. Western blotting and primer extension of Cwc2 and U6 snRNA selected from crosslinked splicing reactions in the presence of Prp2 and Prp2 LAT

(A) Western blotting of Cwc2 crosslinked to the U6 snRNA from three replicate splicing reactions. (B) Primer extension analysis of U6 snRNA selected from three replicate splicing reactions. (C) Levels of U6 snRNA selected in reactions subjected to UV irradiation in Prp2 vs Prp2 LAT supplemented extracts were quantified by phosphorimaging.

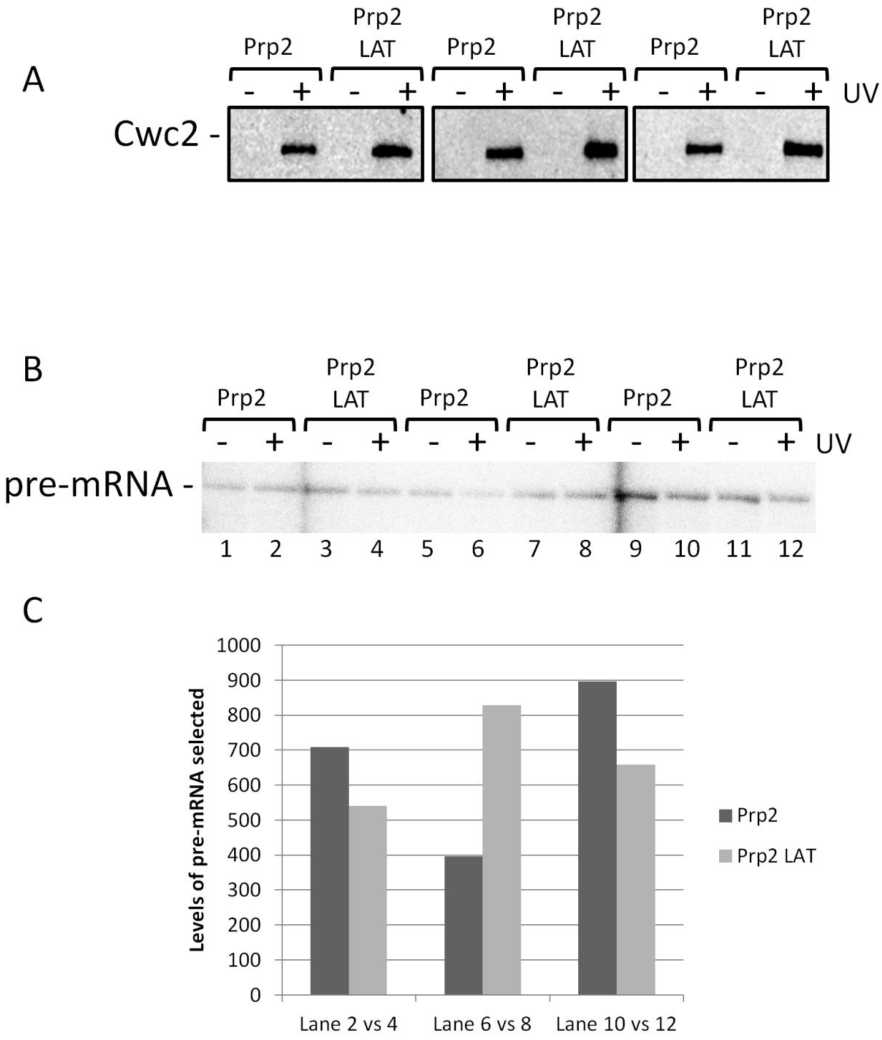

Figure S10. Western blotting and primer extension of Cwc2 and pre-mRNA selected from crosslinked splicing reactions in the presence of Prp2 and Prp2 LAT

(A) Western blotting of Cwc2 crosslinked to the pre-RNA from three replicate splicing reactions. (B) Primer extension analysis of pre-mRNA selected from three replicate splicing reactions. (C) Levels of pre-mRNA selected in reactions subjected to UV irradiation in Prp2 vs Prp2 LAT supplemented extracts were quantified by phosphorimaging.

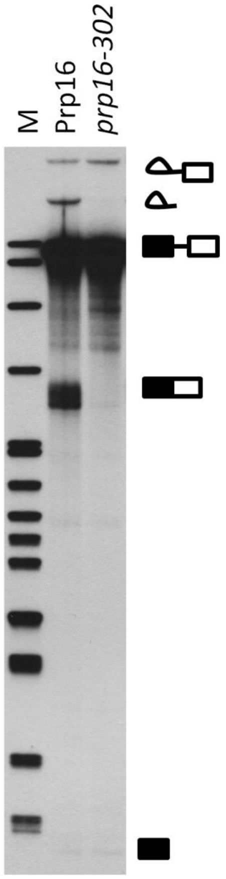

Figure S11. The wild-type Prp16 and *prp16-302* extracts can both assemble spliceosomes capable of splicing

In vitro pre-mRNA splicing of *ACT1* pre-mRNA at 18°C for 55 minutes in yeast whole cells extracts from strains expressing wild-type Prp16 or the *prp16-302* mutation. Both extracts are capable of spliceosome assembly on the pre-mRNA with the Prp16 expressing extract carrying out the two steps of splicing and the *prp16-302* carrying out the first step of splicing, but blocked for the second step.

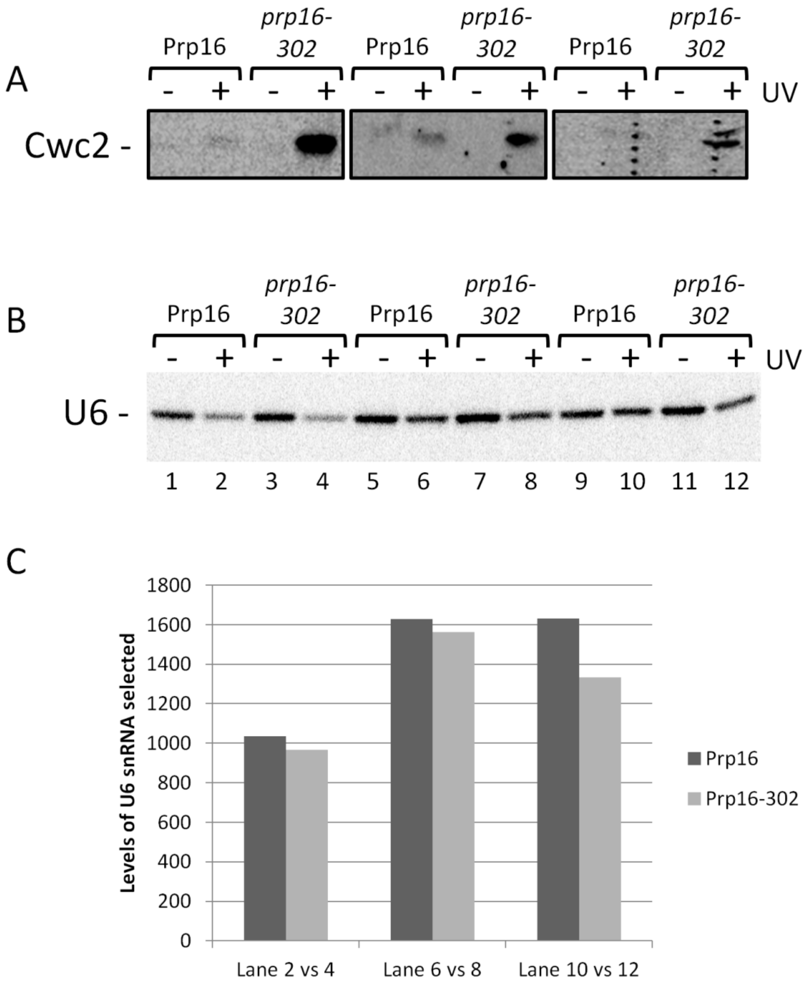

Figure S12. Western blotting and primer extension of Cwc2 and U6 snRNA selected from crosslinked splicing reactions in the presence of Prp16 and *prp16-302*

(A) Western blotting of Cwc2 crosslinked to the U6 snRNA from three replicate splicing reactions. (B) Primer extension analysis of U6 snRNA selected from three replicate splicing reactions. (C) Levels of U6 snRNA selected in reactions subjected to UV irradiation in Prp16 vs *prp16-302* supplemented extracts were quantified by phosphorimaging.

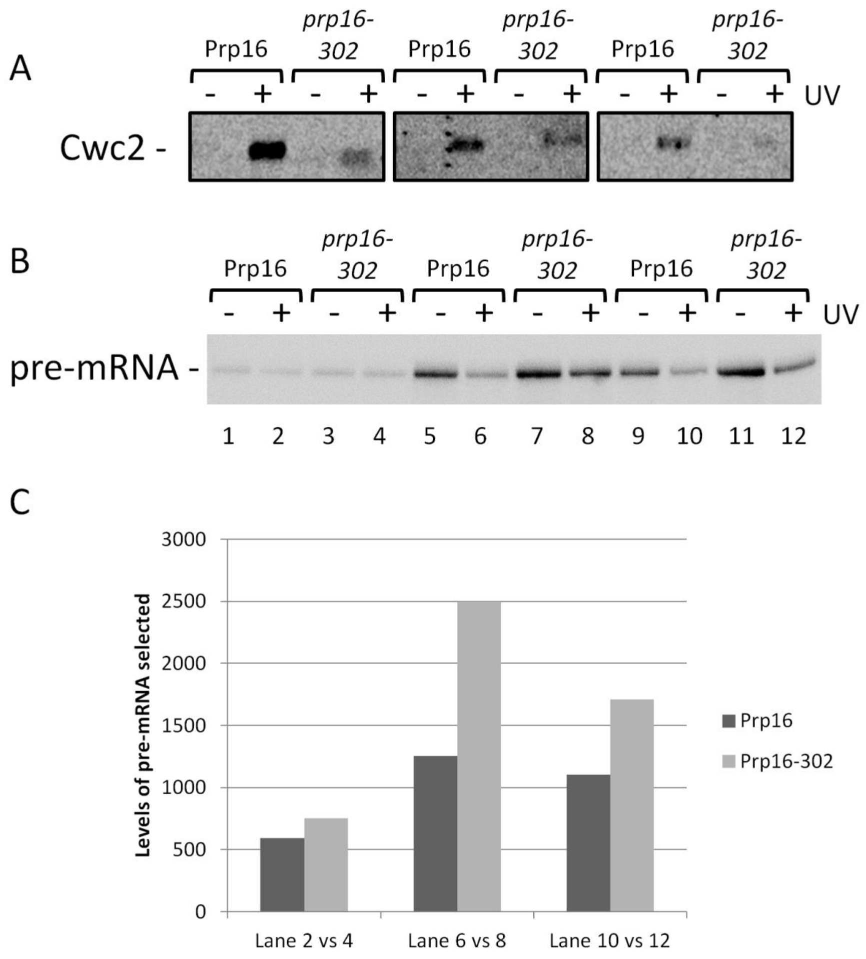

Figure S13. Western blotting and primer extension of Cwc2 and pre-mRNA selected from crosslinked splicing reactions in the presence of Prp16 and *prp16-302*

(A) Western blotting of Cwc2 crosslinked to the pre-mRNA from three replicate splicing reactions. (B) Primer extension analysis of pre-mRNA selected from three replicate splicing reactions. (C) Levels of U6 snRNA selected in reactions subjected to UV irradiation in Prp16 vs *prp16-302* supplemented extracts were quantified by phosphorimaging.

References

Campodonico, E. and Schwer, B. (2002) ATP-dependent remodeling of the spliceosome: intragenic suppressors of release-defective mutants of *Saccharomyces cerevisiae* Prp22. *Genetics*, **160**, 407-415.

Kuhn, A.N., Li, Z. and Brow, D.A. (1999) Splicing factor Prp8 governs U4/U6 RNA unwinding during activation of the spliceosome. *Mol. Cell*, **3**, 65-75.

Li, Z.R. and Brow, D.A. (1996) A spontaneous duplication in U6 spliceosomal RNA uncouples the early and late functions of the ACAGA element *in vivo*. *RNA*, **2**, 879-894.

McGrail, J.C., Krause, A. and O'Keefe, R.T. (2009) The RNA binding protein Cwc2 interacts directly with the U6 snRNA to link the nineteen complex to the spliceosome during pre-mRNA splicing. *Nucleic Acids Res.*, **37**, 4205-4217.

Patterson, B. and Guthrie, C. (1987) An essential yeast snRNA with a U5-like domain is required for splicing *in vivo*. *Cell*, **49**, 613-624.

Schneider, S., Campodonico, E. and Schwer, B. (2004) Motifs IV and V in the DEAH box splicing factor Prp22 are important for RNA unwinding, and helicase-defective Prp22 mutants are suppressed by Prp8. *J. Biol. Chem.*, **279**, 8617-8626.
